# Targeting adenosine 2A receptor signaling suppresses vascular calcification by restraining smooth muscle osteogenic differentiation

**DOI:** 10.1016/j.phrs.2025.108012

**Published:** 2025-10-25

**Authors:** Yaqi Zhou, Dingwei Zhao, Qian Ma, Sujin Lee, Kangsan Roh, Yongfeng Cai, Jiean Xu, Qiuhua Yang, Qingen Da, Zhiping Liu, Kunfu Ouyang, Eric J.Belin de Chantemele, Mei Hong, Clint L. Miller, Rajeev Malhotra, Chunxiang Zhang, Suowen Xu, Yuqing Huo

**Affiliations:** a Department of Physiology, Research Center of Basic Integrative Medicine, School of Basic Medical Sciences, Guangzhou University of Chinese Medicine, Guangzhou 510006, China; b State Key Laboratory of Chemical Oncogenomics, Key Laboratory of Chemical Genomics, School of Chemical Biology and Biotechnology, Peking University Shenzhen Graduate School, Shenzhen 518055, China; c Departments of Ophthalmology, Medicine and Molecular and Cellular Biology, Baylor College of Medicine, Houston, TX 77030, USA; d Cardiovascular Research Center, Division of Cardiology, Department of Medicine, Massachusetts General Hospital, Harvard Medical School, Boston, MA 02114, USA; e Vascular Biology Center, Department of Cellular Biology and Anatomy, Medical College of Georgia, Augusta University, Augusta, GA 30912, USA; f Department of Pharmacological Sciences, Stony Brook University, Stony Brook, NY 11794, USA; g Department of Biochemistry and Molecular Genetics, University of Virginia, Charlottesville, VA 22908, USA; h Center for Public Health Genomics, University of Virginia, Charlottesville, VA 22908, USA; i epartment of Cardiology, Key Laboratory of Medical Electrophysiology, Ministry of Education, Institute of Cardiovascular Research, The Affiliated Hospital of Southwest Medical University, Southwest Medical University, Luzhou 646000, China; j Department of Endocrinology, Centre for Leading Medicine and Advanced Technologies of IHM, The First Affiliated Hospital of USTC, Division of Life Sciences and Medicine, University of Science and Technology of China, Hefei 230001, China; k Anhui Provincial Key Laboratory of Metabolic Health and Panvascular Diseases, Hefei 230001, China; l Institute of Endocrine and Metabolic Diseases, University of Science and Technology of China, Hefei 230001, China

**Keywords:** ADORA2A, CKD, Vascular calcification, Osteogenic differentiation, RUNX2

## Abstract

Vascular calcification (VC) is a major contributor to cardiovascular morbidity and mortality, particularly in patients with chronic kidney disease (CKD). Adenosine 2 A receptor (ADORA2A) is highly expressed in vascular cells and implicated in cardiovascular disease; however, its specific role in VC pathogenesis remains unclear. Here, we investigated the role of ADORA2A using *in vitro* (vascular smooth muscle cells; VSMCs), *ex vivo* (mouse aortic rings), and *in vivo* (5/6th nephrectomy with high phosphate and cholecalciferol) models of VC. The ADORA2A expression was significantly upregulated in calcified human and murine aortic tissues, as well as in VSMCs, under osteogenic conditions. Genetic deletion of *Adora2a* (global or VSMC-specific) or pharmacological antagonism of ADORA2A markedly attenuated aortic calcification and the expression of osteogenic markers *in vivo*. Consistent findings were observed in *in vitro* and *ex vivo* models. Conversely, *ADORA2A* overexpression exacerbated the osteogenic differentiation and calcification of VSMCs. Mechanistically, ADORA2A promoted VSMC osteogenic differentiation by facilitating cAMP-responsive element-binding protein 1 (CREB1) binding to the runt-related transcription factor 2 (*RUNX2*) promoter, thereby enhancing *RUNX2* transcription and subsequent mineralization. Our findings reveal that ADORA2A drives VC through the cAMP/CREB1/RUNX2 signaling axis in VSMCs. Therefore, targeting ADORA2A represents a potential strategy for mitigating VC in CKD.

## 1. Introduction

Cardiovascular disease (CVD) represents the leading cause of mortality in individuals with chronic kidney disease (CKD), with vascular calcification (VC) being a common and independent predictor of cardiovascular events in this population [1]. VC occurs throughout all stages of CKD, resulting in increased vessel wall stiffness, reduced vascular elasticity, and enhanced risk of vascular rupture, which significantly contribute to cardiovascular morbidity and mortality in patients [2]. The prevalence and severity of VC escalate with progressive renal dysfunction [3]. Unfortunately, effective prevention and treatment strategies for VC are hindered by the limited understanding of its pathogenesis.

A pivotal underpinning of VC rests on the osteogenic differentiation of vascular smooth muscle cells (VSMCs), which are typically responsible for maintaining the structure and function of the arterial wall [4,5]. Diverse stimuli, including oxidative stress and elevated phosphate levels, induce VSMCs to undergo dedifferentiation and further osteogenic differentiation [4,5]. This progress entails migration, proliferation, and the secretion of calcifying matrix vesicles and osteogenic proteins, including runt-related transcription factor 2 (RUNX2), alkaline phosphatase (ALPL), and bone morphogenetic proteins (BMPs) [4,5]. The ensuing pathological cascade culminates in extracellular matrix remodeling, elastin degradation, and the establishment of a pro-calcific microenvironment [6,7]. Therefore, elucidating novel targets that effectively control VSMC phenotypic plasticity is crucial for developing new interventions against VC in CKD.

Adenosine is an endogenous purine nucleoside. Adenosine maintains vascular homeostasis under physiological conditions. However, adenosine induces various pathological changes when it accumulates under pathological conditions [8,9]. Adenosine exerts its effects by binding to four G protein-coupled receptors (GPCRs): ADORA1, ADORA2A, ADORA2B, and ADORA3 [9]. Among these, the ADORA2A subtype is abundantly expressed in vascular cells and involved in regulating oxidative burst, inflammation, collagen synthesis, and fibrosis-elements that are intrinsically linked to phenotypic transitions in VSMCs and VC [10–14]. Notably, genome-wide association study (GWAS) has identified a significant association between the ADORA2A locus and coronary artery disease [15]. Furthermore, regarding vascular cell phenotypic transitions, ADORA2A has been demonstrated to be involved in the phenotypic switching of vascular endothelial cells (VECs), playing a critical role in atherosclerotic plaque formation and subretinal fibrosis by modulating endothelial-to-mesenchymal transition (EndMT) [16, 17]. However, the specific contribution of ADORA2A to VSMC osteogenic differentiation and the progression of VC remains enigmatic.

The present study employed a comprehensive set of *in vitro*, *ex vivo*, and *in vivo* VC models to mimic the conditions associated with CKD. We demonstrated that the global deletion of *Adora2a*, VSMC-specific deletion of *Adora2*a, or pharmacological antagonism of ADORA2A effectively inhibited osteogenic differentiation of VSMCs and protected against VC in CKD mice. We further revealed that these effects were mediated by the regulation of *RUNX2* transcription via the cAMP/PKA/CREB1 signaling pathway. Our findings suggest that targeting ADORA2A is a promising strategy to attenuate VC in CKD.

## 2. Materials and methods

### 2.1. Mouse generation and breeding

Global homozygous *Adora2a* (*Adora2a*^−/−^) knockout mice were generated by heterozygous inter-breeding (*Adora2a*^+/−^) as previously described [18], and the wild-type littermates (*Adora2a*^WT^) were used as controls. Breeding pairs of *Adora2a*^Flox/Flox^ (*Adora2a*^F/F^) mice were kindly gifted by Dr. Joel Linden (La Jolla Institute for Allergy and Immunology, CA, USA). *Adora2a*^F/F^ mice were crossbred with *Myh11*^Cre/ERT2^ mice (The Jackson Laboratory; Stock#019079) to generate *Adora2a*^F/F^*Myh11*^Cre/ERT2^ mice, which were injected with tamoxifen to produce VSMC-specific *Adora2a*-deficient (*Adora2a*^ΔVSMC^) mice. All the above mice were on a C57BL/6 J background. Genotyping of these mice was performed using PCR amplification with tail DNA samples. Specific primers used for genotyping were listed in Supplemental Table 1. Female dilute brown non-agouti 2 (DBA/2) mice were purchased from Charles River Laboratories (Sulzfeld, Germany; Stock#214). Animals were housed in groups and maintained under the following conditions: a temperature of 23 ± 2°C, a relative humidity of 60 ± 10 %, and a 12/12 h light/dark cycle, with free access to food and water. A limitation to the generalizability of the study is that it did not consider gender/sex issues.

### 2.2. Tamoxifen injection

Tamoxifen powder (T5648, Sigma, MO, USA) was dissolved in corn oil (C8267, Sigma, MO, USA) to a final concentration of 20 mg/mL by shaking overnight at 37°C, which was injected intraperitoneally into *Adora2a*^F/F^*Myh11*^Cre/ERT2^ mice and their littermate control mice (8-week-old) at a dose of 75 mg/kg body weight for five consecutive days to induce VSMC-specific *Adora2a*-deficient (*Adora2a*^ΔVSMC^) mice. Seven days after the final injection, the mice were used for indicated experiments.

### 2.3. Mouse model of vascular calcification within CKD

The mouse CKD model was established by a 5/6 partial nephrectomy with pole ligation, performed in a two-step surgery [19]. In step-one surgery, mice were first anesthetized with 5 % isoflurane for 3 min and afterward maintained with 2.0 % isoflurane during the surgery. Body temperature was maintained at 37.0 ± 0.5°C using a heating pad during surgery. The mouse was placed on the right side, and the hair in the surgical area was shaved using a hair clipper. After disinfection, a 1.0 cm incision was made in the lumbar region to expose the left kidney. The left kidney was gently pulled out by holding the perinephric fat with blunt forceps. The adrenal gland was carefully moved upwards from the kidney. The ureter was separated from the kidney by gently grasping the connective tissues to avoid ligation. Then, the kidney’s upper third and lower third were ligated using a 3–0 silk suture and with proper force. Following a 2-minute observation period, the poles were discolored due to ischemia. The left kidney was returned to its original place, and the subcutaneous tissues and the skin were sutured. After one week, the step-two surgery was performed. A mouse was placed on the left side, and the right kidney was exposed using the same procedure described above. The renal artery and vein were identified and isolated to tie off with two ligatures (3–0 silk); one was close to the abdominal aorta, and the other was close to the kidney. Then, the vessels were cut between the two knots, and the right kidney was removed, followed by suture and disinfection. Mice in the sham-operation group were only made surgical incisions to expose the kidney without the pole ligation of the left kidney or removal of the right kidney. After recovering, mice were housed in regular sterilized cages with wet food. Meloxicam (1 mg/kg) and cefquinome (5 mg/kg) were administered subcutaneously once daily for three days to prevent pain and infection after each surgery. Kidney function was assessed using serum creatinine levels. Only animals with kidney failure were included in the study.

For the induction of vascular calcification, after one week of recovery, mice were fed with a high phosphate (1.8 %) diet (Guangdong Medical Lab Animal Center, Guangdong, CN), together with cholecalciferol (C9756, Sigma, MO, USA) three times per week by gavage (1 μg/kg) for four weeks. Mice in the sham-operation group were fed a normal phosphate (0.8 %) diet.

### 2.4. Istradefylline (KW6002) administration

A potent ADORA2A antagonist, KW6002, was used to investigate the effect of ADORA2A blockade in vascular calcification. KW6002 was dissolved in DMSO (D2650, Sigma, MO, USA) at a concentration of 20 mg/mL and then mixed with an equal volume of Tween-80 (5 % vol vs 5 % vol). Right before the injection, saline (90 % vol) was added and thoroughly vortexed with the mixture, which was then injected intraperitoneally within 5 min at a final concentration of 10 mg/kg KW6002, once daily for four weeks, along with the high phosphate diet supplement. This dose of KW6002 was based on previous studies and is effective in specifically antagonizing the ADORA2A-mediated effects in rodent disease models [17,18,20,21].

### 2.5. Animal experiments

The animal experiments in this study were divided into three parts. To determine the role of ADORA2A in vascular calcification, male *Adora2a*^−/−^ mice and their littermates aged 9–10 weeks were randomly grouped as follows: *Adora2a*^WT^+sham (n = 14); *Adora2a*^WT^+CKD (n = 30); *Adora2a*^−/−^+sham (n = 14); *Adora2a*^−/−^+CKD (n = 30). To determine the effect of VSMC-specific deficiency of *Adora2a* in vascular calcification, male *Adora2a*^ΔVSMC^ mice and their littermates aged 9–10 weeks were randomly separated into three groups: *Myh11*^Cre/ERT2^+-Sham (n = 6) *Myh11*^Cre/ERT2^+CKD (n = 30); *Adora2a*^ΔVSMC^+CKD (n = 30); For the *in vivo* experiment of KW6002, female DBA/2 mice aged 9–10 weeks were randomly divided into three groups: sham with vehicle (n = 20); CKD with vehicle (n = 30); CKD with KW6002 (n = 30). The animal numbers required in each experiment to achieve statistical significance were determined based on prior experience from the literature. Researchers for the outcome evaluations were blind to the treatment assignment. Experimental details, including control and treatment groups, sample sizes, and statistical analyses employed, are provided in the accompanying figure legends.

### 2.6. Ex vivo aortic ring culture

The osteogenic medium was prepared as follows: DMEM (11965092, Thermo Fisher Scientific, MA, USA) supplemented with 1 % fetal bovine serum (FBS), 1 % Antibiotic-Antimycotic (15240062, Thermo Fisher Scientific, MA, USA), 10 mM β-glycerophosphate disodium (G9422, Sigma, MO, USA), 0.25 mM L-ascorbic acid (49752, Sigma, MO, USA), and 10 nM dexamethasone (D4902, Sigma, MO, USA). The control medium was prepared with DMEM supplemented with 1 % FBS and 1 % Antibiotic-Antimycotic.

After the mice were euthanized, the aortas were carefully dissected after *in situ* perfusion with sterile PBS. Descending aortas were collected, cut into 2 mm rings, and cultured in either an osteogenic medium or a control medium for 2 weeks in a humidified incubator with 5 % CO_2_ at 37°C. The medium was changed every 2–3 days [22,23]. At the end of the experiments, aortic rings were harvested for the indicated experiments.

### 2.7. Determination of arterial calcification

The presence of arterial calcification was determined by alizarin red and von Kossa staining. Mouse aortic arteries were fixed in 95 % ethanol for 24 h and then stained with 0.003 % alizarin red solution (A5533, Sigma, MO, USA) in 1 % potassium hydroxide overnight for the whole mount of aorta staining. The mouse arterial tissues were then rinsed in 2 % potassium hydroxide three times before taking photos. For the cross-section staining, human tibial arteries were embedded in paraffin and sectioned at 8 mm intervals. Mouse aortic segments were fixed in 4 % paraformaldehyde, embedded in OCT, and cut into 6 μm thick sections. For alizarin red staining, human paraffin sections were deparaffinized, rehydrated, and stained with 1 % alizarin red S solution (A5533, Sigma, MO, USA). The mouse frozen sections were stained with a 0.2 % alizarin red solution (pH 8.3) (C0140-100mL, Beyotime, Shanghai, CN) for 20 min at room temperature and then rinsed with deionized water to remove the excess dye. For von Kossa staining, the mouse frozen sections were incubated with 1 % silver nitrate solution (GMS80045.3, Genmed, Shanghai, CN) in front of a 60-watt light bulb for 45 min. Unreacted silver was removed with 5 % sodium thiosulfate for 5 min, and the sections were counterstained with nuclear fast red (GMS40011, Genmed, Shanghai, CN) according to the manufacturer’s protocol [24, 25]. Images were captured under an inverted microscope.

To assess the deposited calcium, descending aortas were separated and lyophilized. After being weighed, the mouse aortas were decalcified for 48 h using 0.6 mM HCl at 37°C. The calcium concentration in the HCl supernatant was determined colorimetrically using the QuantiChrom^™^ Calcium Assay Kit (DICA-500, BioAssay Systems, CA, USA). The amount of vascular calcium was normalized to the dry weight of the aortas and expressed as fold change compared to the corresponding control.

### 2.8. Serum biochemistry

The whole blood was collected and allowed to clot at room temperature. The serum was separated by centrifugation at 2000 × *g* for 10 min. The creatinine, phosphorus, and calcium levels were measured by a spectrophotometer with commercially available kits (E-BC-K188-M, E-BC-K245-M, E-BC-K103-M, respectively; Elabscience, Wuhan, CN) according to the manufacturer’s instructions.

### 2.9. Alkaline phosphatase (ALPL) activity assay

ALPL activity was measured using a commercially available kit (P0321S, Beyotime, Shanghai, China) according to the manufacturer’s instructions. Briefly, cells or aortic tissues were lysed in cell lysis buffer without inhibitors (P0013J, Beyotime, Shanghai, China). After centrifugation, the supernatant was reacted with the color-developing substrate for 10 min at 37 °C and followed by incubation with the stop solution. The absorbance of samples was measured at 405 nm.

### 2.10. Immunofluorescence and microscopy

Human tibial arteries were embedded in paraffin and cry-sectioned at a thickness of 8 mm. Sections were deparaffinized and rehydrated. Subsequently, sections were incubated with primary antibodies specific to ADORA2A (0.69 mg/mL, PA1-042, Thermo Fisher Scientific, MA, USA) or ACTA2 (12.3 μg/mL, F3777, Sigma, MO, USA), and secondary antibody Cy^™^3 AffiniPure Donkey anti-Rabbit IgG (H+L) (711-165-152, Jackson ImmunoResearch, PA, USA). Images were obtained with a spinning disk confocal microscope (SoRa, Nikon).

Mouse aortic tissues were embedded in OCT and cry-sectioned at a thickness of 6 μm. Sections were fixed with 4 % paraformaldehyde for 15 min at room temperature, permeabilized with 0.5 % Triton X-100 in PBS for 30 min, and blocked with 10 % normal goat serum for 1 h. Subsequently, sections were incubated with primary antibodies specific to ADORA2A (5 μg/mL, 05–717, Sigma, MO, USA) or TAGLN (2 μg/mL, 14106, Abcam, Cambridge, UK) overnight at 4°C, and a mixture of Alexa Fluor 594 goat anti-mouse IgG (4 μg/mL, A11032, Invitrogen, CA, USA) and Alexa Fluor 488 goat anti-rabbit IgG (4 μg/mL, A11034, Invitrogen, CA, USA) for 1 h at room temperature. Nuclear staining was performed using DAPI (10236276001, Roche, Mannheim, Germany). Slides were mounted with an anti-fade medium (H-1000, Vector Laboratories, CA, USA) for subsequent confocal microscope (Nikon).

### 2.11. Histology and immunohistochemistry (IHC)

The pathological changes in the thoracic aortas were examined by hematoxylin and eosin (HE) staining. Mouse aortic Section (6-μm thick) were stained with hematoxylin for 5 min, differentiated with 1 % hydrochloric acid alcohol, and then stained with eosin for 1 min. Following dehydration and drying, the sections were sealed with neutral gum and subjected to digital scanning and analysis.

To detect BMP2 expression, IHC was performed by using a ready-to-use high-potency IHC secondary antibody kit (abs957, Absin, Shanghai, CN) according to the manufacturer’s instructions. Briefly, mouse aortic Section (6-μm thick) were fixed in cold acetone and incubated with hydrogen peroxide for 10 min, and followed by a blocking buffer for 5 min. Primary antibody specific to BMP2 (3.2 μg/mL, ab284387, Abcam, Cambridge, UK) was added at room temperature for 20 min. After incubating with the direct antibody amplifier for 10 min and the HRP-conjugated secondary antibody polymer for 10 min, a fresh substrate solution was applied to the sections and incubated for 5 min. Following dehydration and drying, the sections were sealed with neutral gum and subjected to digital scanning and analysis.

### 2.12. Cell culture and treatment

Primary human aortic smooth muscle cells (HASMCs) were purchased from American Type Culture Collection (PCS-100–012, ATCC, VA, USA) and cultured in smooth muscle cell medium (SMCM, 1101, ScienCell, CA, USA) in a humidified atmosphere with 5 % CO_2_ at 37°C. The medium was changed every other day until the culture was approximately 80 % confluent. All *in vitro* experiments were performed with cells at passages 4–6.

Primary mouse aortic smooth muscle cells (MASMCs) were isolated from 3-week-old male mice. After the mice were euthanized, the aortas were carefully dissected after *in situ* perfusion with sterile PBS. Descending aortas were collected, washed three times with PBS, and placed into the HBSS (24020-117, Gibco, NY, USA) containing collagenase II (1 mg/mL, LS004176, Worthington, NJ, USA), elastase (0.744 units/mL, LS002279, Worthington, NJ, USA), and Soybean Trypsin Inhibitor (1 mg/mL, 17075-029, Gibco, NY, USA) for 8 min in cell culture incubator. Then, the adventitia was stripped off, and the aortas were placed back into the enzyme solution for another 60 min, or until the vascular structure had disappeared and the cells appeared as single cells or strings. The enzyme was inactivated with culture medium DMEM (SH30021.01, Hyclone, DE, USA) supplemented with 20 % FBS and 1 % Antibiotic-Antimycotic. The cell suspension was collected into a tube and centrifuged for 8 min at 1000 rpm. The pellet was suspended in a culture medium and plated at a density depending on cell recovery. The cells were cultured in a humidified incubator with 5 % CO_2_ at 37°C.

### 2.13. Osteogenic induction

Cells were cultured in growth medium until full confluence, and then switched to osteogenic medium (OM, DMEM containing 10 % FBS, 1 % Antibiotic-Antimycotic, 10 mM β-glycerophosphate disodium, 0.25 mM L-ascorbic acid, and 10 nM dexamethasone) or control medium (CM, DMEM containing 10 % FBS, 1 % Antibiotic-Antimycotic) for the indicated time. During the induction process, the medium was replaced every three days.

Cell calcification was determined by alizarin red staining and von Kossa staining. Cells were fixed with 4 % paraformaldehyde and then washed with deionized water. For alizarin red staining, cells were stained with 2 % alizarin red solution (pH 4.2) (ECM-815, Sigma, MO, USA) for 10 min at room temperature and then rinsed with deionized water. For von Kossa staining, cells were incubated with a 1 % silver nitrate solution (GMS80045.3, Genmed, Shanghai, CN) in the presence of a 60-watt light bulb until the solution turned black. Plates were washed with 5 % sodium thiosulfate for 5 min to remove unreacted silver. Images were captured under an inverted microscope. Representative images were selected to represent the mean value of each condition.

### 2.14. RNA interference

Cells were seeded in 6-well plates and grown in a growth medium to reach 60–70 % confluence. HASMCs were transfected with 50 nM siRNAs targeting human *ADORA2A* (si*ADORA2A*, sc-39850, Santa Cruz Biotechnology, TX, USA; si*RUNX2*, sc-37145, Santa Cruz Biotechnology, TX, USA; si*CREB1*, sc-29281, Santa Cruz Biotechnology, TX, USA) or non-targeting negative control (si*CTRL* for si*ADORA2A*, si*RUNX2* and si*CREB1*, sc-37007, Santa Cruz Biotechnology, TX, USA) using Lipofectamine RNAiMAX Reagent (13778-150, Invitrogen, CA, USA) according to the manufacturer’s protocol. Six hours after transfection, the medium was replaced with fresh growth medium, followed by continuous culture for 24 h before the indicated experiments.

### 2.15. Adenovirus transduction of HASMCs

HASMCs grown at 70 %–80 % confluence were incubated with 1 mL smooth muscle cell basal medium media containing control or *ADORA2A* adenovirus. After 1 h, the medium was changed to the fresh SMC medium for an additional culture. At the indicated time points, cells were collected for analysis.

### 2.16. Quantitative PCR (qPCR) analysis

The total RNA of cells was extracted with TRIzol reagent (15596018, Invitrogen, CA, USA) according to the manufacturer’s instructions [26, 27]. 1 μg of total RNA was reverse-transcribed using TransScript^®^ One-Step gDNA Removal and cDNA Synthesis SuperMix kit (AT311, TransGen Biotech, Beijing, CN). Real-time PCR was performed using a CFX96 instrument (Bio-Rad) or a QuantStudio 5 Real-Time PCR System (Applied Biosystems) with universal SYBR Green mix (AQ601, TransGen Biotech, Beijing, China). Gene-specific primers used for PCR are shown in Supplemental Table 2. Quantification of relative gene expression was calculated using the 2^−ΔΔCT^ method, with *18S* ribosomal RNA or *ACTA* as the internal control. The data were presented as a fold change relative to the control groups.

### 2.17. Western blot analysis

Cultured cells or aorta samples were lysed in RIPA buffer (S0278, Sigma, MO, USA) with 1 % protease inhibitors (4693159001, Roche, Mannheim, Germany), 1 % phosphatase inhibitors (4906845001, Roche, Mannheim, Germany), and 1 % PMSF (ST506, Beyotime, Shanghai, CN) on ice. Protein concentration was quantified using the BCA Protein Assay kit (23225, Thermo Fisher Scientific, MA, USA). Equal amounts of protein per lane (10 μg) were subjected to 8–12 % SDS-PAGE. Antibodies used in this study were provided in Supplemental Table 3. Immunoreactive proteins were detected by the chemiluminescence assay (Millipore) using the ChemiDoc MP system (Bio-Rad) or e-Blot Touch Imager (e-Blot). Quantification was performed using ImageJ, a free software, and each lane was normalized to GAPDH.

### 2.18. Chromatin immunoprecipitation (ChIP) and ChIP-qPCR analysis

A ChIP assay was performed using the Pierce Agarose ChIP Kit (26156, Thermo Fisher Scientific, MA, USA) according to the manufacturer’s instructions. Briefly, cultured cells were crosslinked with 1 % formaldehyde for 10 min, inactivated with 1 × glycine for 5 min at room temperature, and collected in ice-cold PBS with 1 × Halt Cocktail. Cell pellets were resuspended and incubated in Lysis Buffer 1 containing protease inhibitors on ice for 10 min. Nuclei pellets were spinned down at 9000 × *g* for 3 min and resuspended in MNase Digestion Buffer Working Solution with Micrococcal Nuclease (ChIP Grade), and then incubated in a 37°C water bath for 15 min. MNase Stop Solution was added and incubated on ice for 5 min to stop the reaction. The mixture was centrifuged at 9000 × *g* for 5 min to recover the nuclei pellets, which were then resuspended in Lysis Buffer 2 containing protease/phosphatase inhibitors and incubated on ice for 15 min. After centrifugation at 9000 × *g* for 5 min, the supernatant containing the digested chromatin fragments was transferred to a new 1.5 mL tube to mix with 1 × IP Dilution Buffer and incubated with the corresponding primary antibody for 16 h at 4°C. Then, the IP reactions were incubated with ChIP Grade Protein A/G Plus Agarose beads for 1 h at 4°C. Beads were washed three times with IP wash buffer. The antibody/protein/DNA complexes were eluted with IP Elution buffer and incubated with NaCl and Proteinase K at 65°C for 1.5 h. Then, DNA was purified and eluted with 50 μL DNA Column Elution Solution.

DNA samples were analyzed by qPCR to detect the binding to *RUNX2* promoter using primer specific for the *RUNX2* promoter (Forward: TGCAGCTGGGTAGCA, Reverse: TGTGTTTGGCCTGGGGA). 2 μL of DNA and 100 nM of primers were used in 20 μL of reaction volume. ChIP-qPCR enrichment of target loci was normalized to input DNA.

### 2.19. Statistical analysis

Data were represented as mean ± SEM. Comparisons between the two groups were performed using a nonparametric Mann-Whitney *U*-test when the data were not normally distributed, an unpaired Student’s t-test followed by Welch’s correction when the data passed normality but not variance test, and an unpaired Student’s *t*-test when the data passed both normality and variance test. Multiple comparisons were performed by nonparametric Kruskal-Wallis test followed by Dunn’s *post hoc* test when the data was not normally distributed, Brown-Forsythe and Welch’s ANOVA test with Dunnett’s T3 multiple comparison test when the data passed normality but not variance test, and one-way analysis of ANOVA followed by Bonferroni’s *post hoc* test when the data passed both normality and variance test. Statistical tests used for each experiment were specified in figure legends. All statistical analyses were performed using the GraphPad Prism software (Version 9.0). No outliers were removed from the data. *p* < 0.05 was considered significant (*p < 0.05, **p < 0.01, ***p < 0.001). “ns” indicates no significant difference. All data shown are representative of at least three independent experiments.

## 3. Results

### 3.1. ADORA2A is positively correlated with VC in both human and mouse specimens

**Fig. 1.**
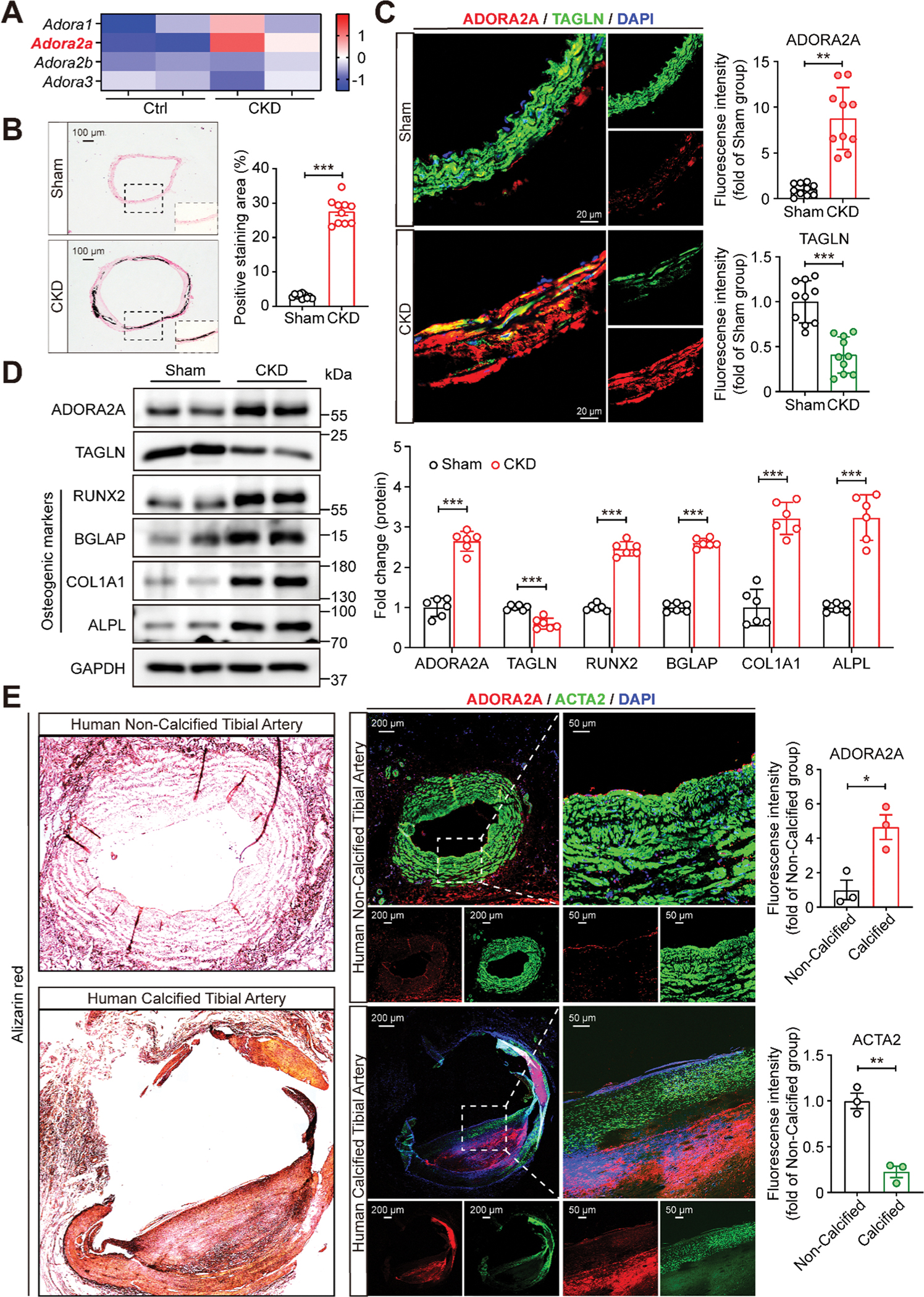
ADORA2A is upregulated in calcified aortic tissues from mice and humans. (A) Heat map showing the mRNA levels of adenosine receptors in the aortas of control and CKD mice. (B) Representative images of von Kossa-stained aortic sections of sham and CKD mice (n = 10). (C) Representative images of immunofluorescence staining and quantification of ADORA2A (red) and TAGLN (green) expression in the thoracic aortas of sham and CKD mice. The nucleus (blue) was stained with DAPI (n = 10). (D) Western blot analysis and quantification of ADORA2A and osteogenic markers in thoracic aortas of sham and CKD mice (n = 6). (E) Representative images of alizarin red-stained aortic sections and immunofluorescence staining of ADORA2A (red) and ACTA2 (green) expression in the human ischemic tibial arteries. Tibial arteries from patients undergoing lower extremity amputation due to critical limb-threatening ischemia. Control arteries were obtained from patients undergoing amputation during orthopedic reconstruction without a prior history of peripheral artery disease or lower extremity ischemia. The nucleus (blue) was stained with DAPI (n = 3). Data are represented as means ± SEM. Statistical significance was determined by unpaired two-tailed Student’s *t-test*. *p < 0.05, **p < 0.01, and ***p < 0.001 for indicated comparisons.

To explore the potential relevance of ADORA2A to VC in CKD, we initially reanalyzed a publicly available microarray dataset derived from murine aortas (Gene Expression Omnibus (GEO) database, accession GSE159833) [28]. We compared mRNA expression of adenosine receptors in aortic tissue between mice with CKD and control mice. As shown in Fig. 1A and Supplemental Table 4, aortic *Adora2a* mRNA levels were significantly elevated in mice with CKD compared to control mice. However, mRNA expressions of other adenosine receptors, specifically *Adora2b* and *Adora3*, did not significantly differ between the groups, whereas *Adora1* mRNA levels exhibited considerable intra-group variability. To confirm this observation, a murine model of CKD-associated VC was established. Mice underwent a 5/6th subtotal nephrectomy via pole ligation and were subsequently maintained on a high-phosphate diet supplemented with cholecalciferol (Supplementary material online, Figure S1A). Compared to sham-operated controls, mice with CKD exhibited significantly reduced survival and greater body weight loss following the induction of CKD (Supplementary material online, Figure S1B and S1C). Serum creatinine and phosphorus levels were robustly elevated in CKD mice, whereas serum calcium levels did not differ distinctly between sham and CKD mice (Supplementary material online, Figure S1D). Despite normocalcemia, aortic calcium deposition was significantly increased in CKD mice, as determined by quantitative aortic calcium content analysis and whole-mount aorta staining (Supplementary material online, Figures S1E and S1F), collectively confirming the successful establishment of VC. Subsequently, we evaluated the expression of ADORA2A in these calcified murine aortas. Corresponding with increased calcification of arteries shown by von Kossa staining, immunostaining of aortic sections demonstrated increased expression of ADORA2A in the aortas of CKD mice compared with that in the sham group (Figs. 1B and 1C). Concurrently, the medial thickness was increased, VSMC marker smooth muscle protein α (TAGLN) was markedly reduced, and the osteogenic marker BMP2 was upregulated (Fig. 1C and Supplementary material online, Figure S1G). Western blots of lysates from aortas of CKD mice showed elevated ADORA2A protein levels, accompanied by the upregulation of osteogenic markers (Fig. 1D).

To further study the expression of ADORA2A in human calcified arteries, tibial artery specimens were procured from patients undergoing below-knee amputation. Patients were categorized based on the presence of critical limb ischemia (CLI) or served as a control group, undergoing amputation for non-ischemic clinical indications. Alizarin red staining revealed significantly increased calcification in the tibial arteries of patients with CLI compared to those in control subjects. This heightened arterial calcification was paralleled by considerably elevated ADORA2A protein expression, as determined by immunofluorescence analysis (Fig. 1E). In parallel, primary human aortic SMCs (HASMCs) cultured in osteogenic medium (OM) were employed as an *in vitro* model of VC. As shown in Supplementary material online, Figure S2, HASMCs cultured in OM exhibited robust mineralization, which was associated with upregulated expression of osteogenic markers and downregulated expression of SMC contractile markers, compared to cells cultured in control medium (CM). Notably, ADORA2A mRNA and protein levels were also markedly elevated in HASMCs under osteogenic conditions. Collectively, these findings suggest a potential involvement of ADORA2A in the pathogenesis of VC.

### 3.2. ADORA2A regulates the osteogenic differentiation of cultured VSMCs

**Fig. 2.**
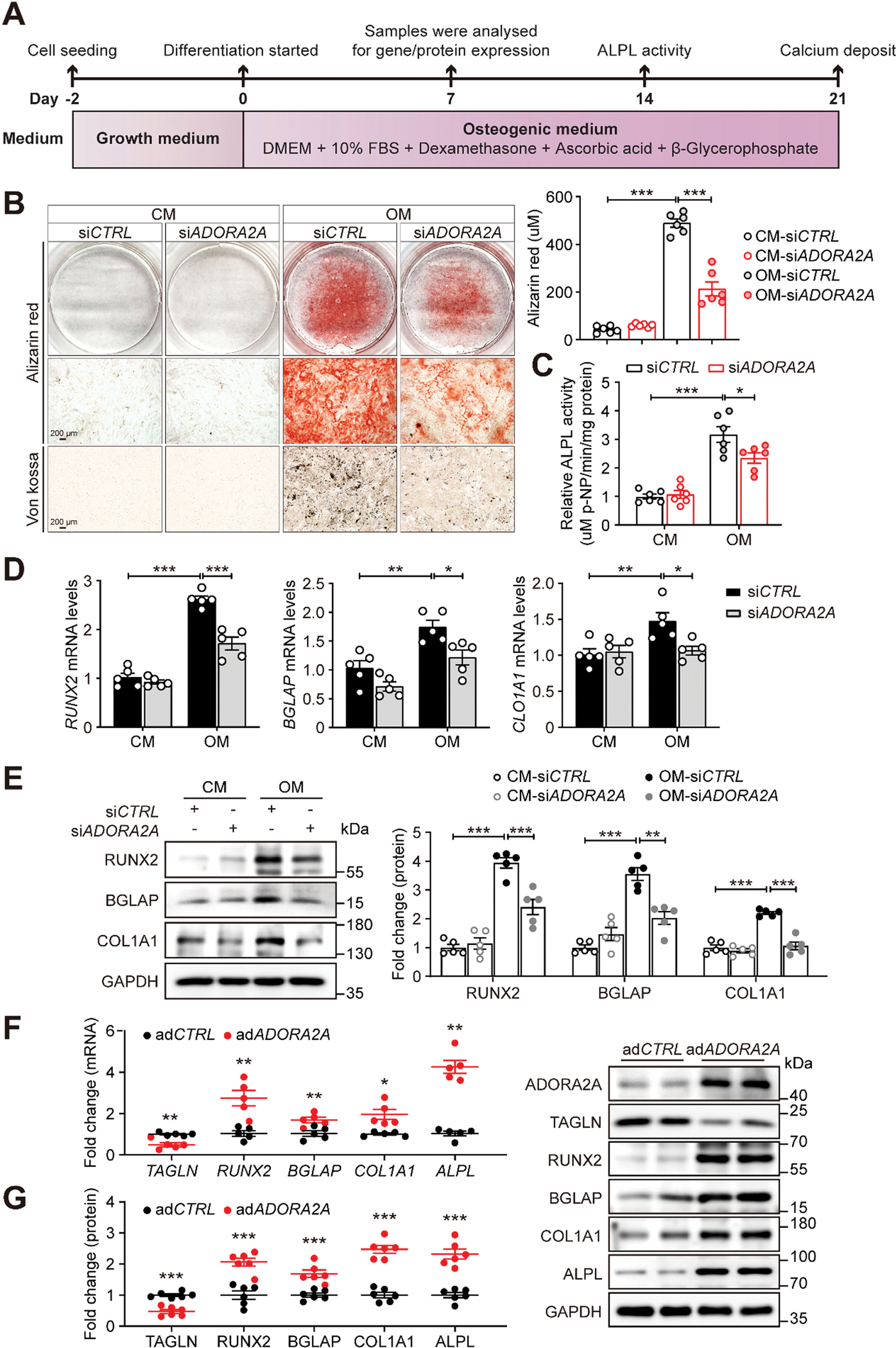
*ADORA2A* knockdown prevents osteogenic differentiation and calcification of HASMCs. (A) Schematic of the experimental design for *in vitro* SMC calcification. (B) Representative images and quantification of alizarin red-stained HASMCs transfected with control or *ADORA2A* siRNA and exposed to OM for 21 days (n = 6). (C) Quantification data of ALPL activity for HASMCs transfected with control or *ADORA2A* siRNA and exposed to OM for 14 days (n = 6). (D) qPCR analysis of mRNA levels of indicated genes in HASMCs transfected with control or *ADORA2A* siRNA and exposed to OM for 7 days (n = 5). (E) Western blot analysis and quantification of indicated protein expression in HASMCs transfected with control or *ADORA2A* siRNA and exposed to OM for 7 days (n = 5). (F) qPCR analysis of mRNA levels of indicated genes in HASMCs infected with control or *ADORA2A* adenovirus for 5 days (n = 5). (G) Western blot analysis and quantification of indicated proteins in HASMCs infected with control or *ADORA2A* adenovirus for 5 days (n = 6). Data are represented as means ± SEM. Statistical significance was determined by one-way ANOVA with the Bonferroni’s *post hoc* test (B-E) and unpaired two-tailed Student’s *t-test* (F and G). *p < 0.05, **p < 0.01, and ***p < 0.001 for indicated comparisons.

It is well established that the osteogenic differentiation of VSMCs plays a central role in the development of VC. To examine the role of ADORA2A in this process, ADORA2A expression was silenced in HASMCs using specific siRNA (Supplementary material online, Figure S3). ADORA2A silencing significantly reduced OM-enhanced calcium deposition and ALPL activity (Fig. 2A–C). Furthermore, *ADORA2A* knockdown reduced the OM-induced upregulation of osteogenic markers RUNX2, BGLAP, and COL1A1 at both mRNA and protein levels (Fig. 2D and E). To corroborate these loss-of-function findings in human cells, primary mouse aortic SMCs (MASMCs) were isolated from *Adora2a*^−/−^ mice and their wild-type *Adora2a*^WT^ littermates. The absence of ADORA2A protein was confirmed via western blot analysis, in contrast to that of *Adora2a*^WT^ controls. Under physiological conditions, *Adora2a* deficiency did not alter BMPs signaling pathway activity in MASMCs (Supplementary material online, Figure S4). However, consistent with observations in HASMCs, osteogenic differentiation and calcium deposition were mitigated in murine *Adora2a*^−/−^ SMCs compared with those of *Adora2a*^WT^ SMCs following OM incubation (Supplementary material online, Figure S5). Additionally, complementary gain-of-function studies with *ADORA2A* overexpression in HASMCs were also conducted. Conversely, *ADORA2A* overexpression with an adenoviral vector (ad*ADORA2A*) resulted in decreased expression of the contractile marker TAGLN, alongside increased expression of osteogenic markers (Figs. 2F and 2G). Collectively, these *in vitro* data indicate that ADORA2A modulates the osteogenic phenotypic switch of VSMCs.

### 3.3. VSMC-specific deletion of Adora2a alleviates VC in CKD mice

**Fig. 3.**
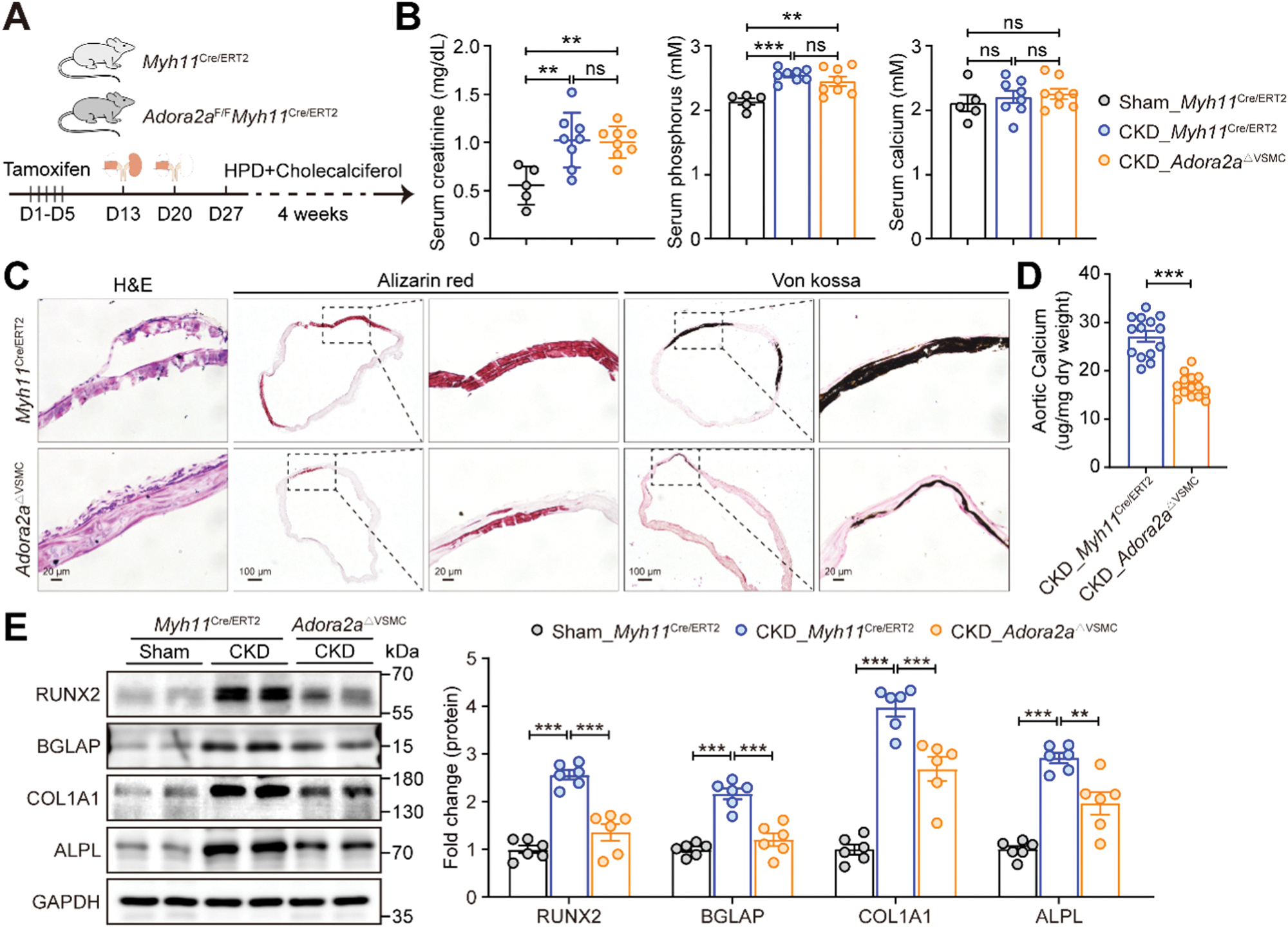
VSMC-specific *Adora2a* deficiency attenuates VC in CKD mice. (A) Schematic of the experimental design for the CKD-associated vascular calcification model in *Myh11*^Cre/ERT2^ and *Adora2a*^ΔVSMC^ mice. (B) Serum concentration of creatinine, phosphorus, and calcium of sham and CKD mice (n = 8). (C) Representative images of alizarin red-, von Kossa-, and HE-stained aortic sections of *Myh11*^Cre/ERT2^ and *Adora2a*^ΔVSMC^ mice (n = 8). (D) Total calcium content in the descending aortas of *Myh11*^Cre/ERT2^ and *Adora2a*^ΔVSMC^ mice. Results shown are normalized by dry weight (n = 14). (E) Western blot analysis and quantification of osteogenic markers in thoracic aortas of *Myh11*^Cre/ERT2^ and *Adora2a*^ΔVSMC^ mice (n = 6). Data are represented as means ± SEM. Statistical significance was determined by one-way ANOVA with the Bonferroni’s *post hoc* test (B, E) and unpaired two-tailed Student’s *t-test* (D). **p < 0.01, and ***p < 0.001 for indicated comparisons. “ns” indicates no significant difference.

To investigate the role of ADORA2A in VSMCs *in vivo*, we generated VSMC-specific *Adora2a-deficient* (*Adora2a*^ΔVSMC^) mice by cross-breeding the *Adora2a*^F/F^ mice with *Myh11*^Cre/ERT2^ mice. The efficiency of *Adora2a* knockout in the medial layers of mouse aortas was demonstrated by Western blot analysis (Supplementary material online, Figure S6). Subsequently, *Adora2a*^ΔVSMC^ mice and their wild-type control mice underwent 5/6th partial nephrectomy and were placed on a high phosphate diet with cholecalciferol supplementation (Fig. 3A). Serum chemistry analysis revealed significantly higher serum creatinine and phosphorus levels in CKD mice compared to sham-operated mice, with no significant differences between *Adora2a*^ΔVSMC^ mice and their controls (Fig. 3B). However, serum calcium levels did not differ significantly between sham and CKD mice, nor between *Adora2a*^ΔVSMC^ and control CKD mice (Fig. 3B). Aortic calcium deposition and osteogenic differentiation were then assessed. As shown in Fig. 3C, calcium deposition in the aortas visualized with alizarin red and von Kossa staining was strikingly reduced in *Adora2a*^ΔVSMC^ mice compared with that in control *Myh11*^Cre/ERT2^ mice. A similar change was observed in the measurement of calcium content in mouse aortas (Fig. 3D). This decreased calcification was associated with diminished expression of osteogenic markers, including RUNX2, BGLAP, COL1A1, and ALPL, in the calcified aortas of *Adora2a*^ΔVSMC^ mice compared with controls (Fig. 3E). Taken together, ADORA2A in VSMCs contributes to the development of VC under the conditions of CKD.

### 3.4. CREB1 is required for ADORA2A-mediated osteogenic differentiation of VSMCs

**Fig. 4.**
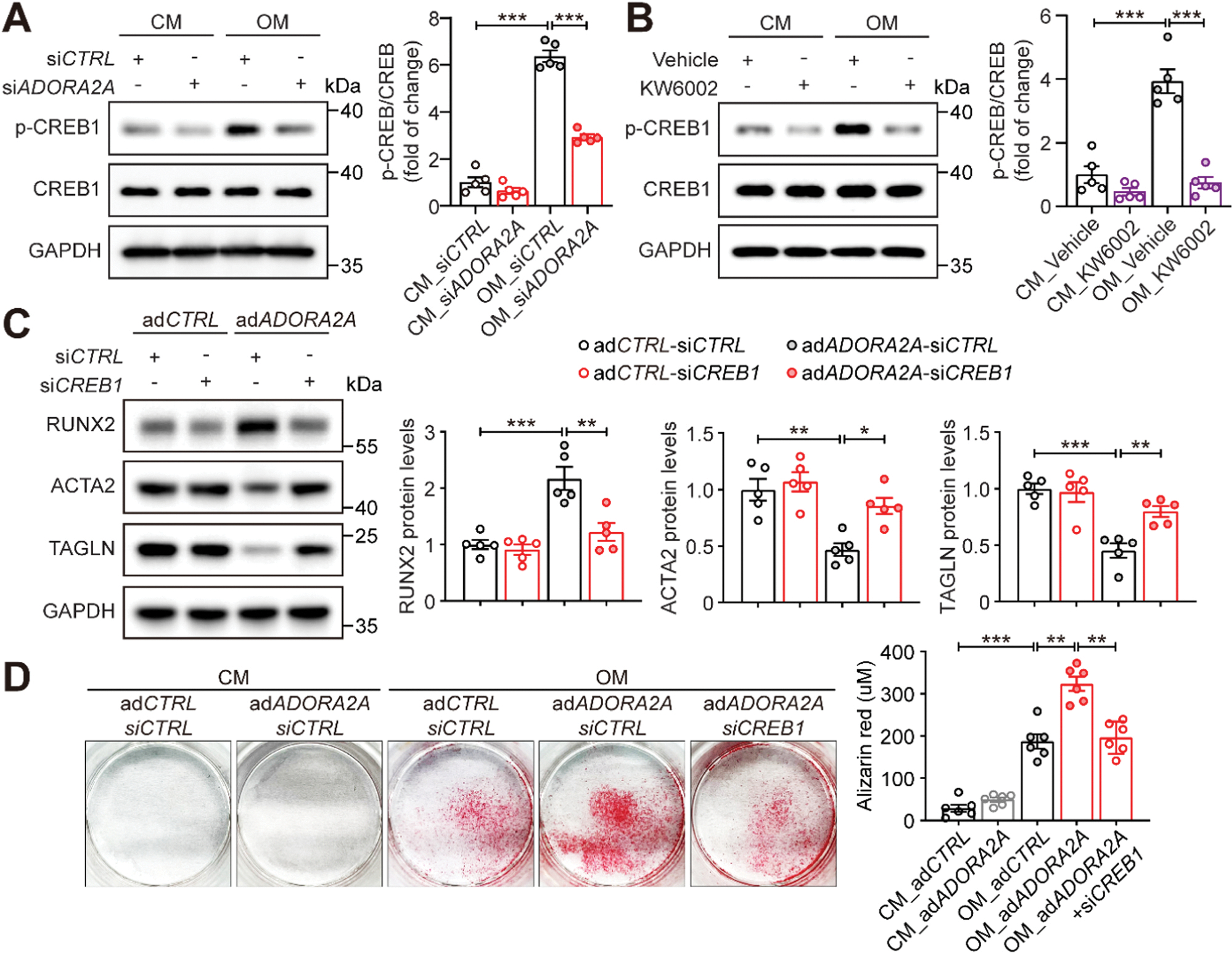
Essential role of CREB1 in ADORA2A-driven osteogenic differentiation of VSMCs. (A) Western blot analysis and quantification of p-CREB1 and CREB1 protein expression in HASMCs transfected with control or *ADORA2A* siRNA and exposed to OM for 5 days (n = 5). (B) Western blot analysis and quantification data of p-CREB1 and CREB1 protein expression in HASMCs treated with vehicle or KW6002 and exposed to OM for 5 days (n = 5). (C) Western blot analysis and quantification of indicated protein expression in HASMCs transfected with control or *CREB1* siRNA and infected with control or *ADORA2A* adenovirus for 5 days (n = 5). (D) Representative images and quantification of alizarin red-stained HASMCs transfected with control or *CREB1* siRNA and infected with control or *ADORA2A* adenovirus, followed by OM treatment for 21 days (n = 6). Data are represented as means ± SEM. Statistical significance was determined by one-way ANOVA with the Bonferroni’s *post hoc* test. *p < 0.05, **p < 0.01, and ***p < 0.001 for indicated comparisons.

As a typical GPCR, ADORA2A participates in many physio- and pathological processes through the cAMP/PKA/CREB1 signaling pathway. To elucidate the mechanism by which ADORA2A regulates vascular calcification, we first assessed whether OM treatment affected the activity of this pathway. As shown in Figs. 4A and 4B, CREB1 was more prominently phosphorylated in VSMCs treated with OM compared to cells treated with CM; this increased CREB1 phosphorylation was attenuated by *ADORA2A* knockdown or treatment with the ADORA2A antagonist istradefylline (KW6002), suggesting potential CREB1 involvement in ADORA2A-mediated osteogenic differentiation of VSMCs. To confirm this hypothesis, *CREB1* expression was silenced using specific siRNA (si*CREB1*) in VSMCs engineered to overexpress *ADORA2A*. *CREB1* silencing effectively antagonized the ad*ADORA2A*-induced upregulation of the osteogenic marker RUNX2 and the downregulation of contractile markers ACTA2 and TAGLN (Fig. 4C). Furthermore, *ADORA2A* overexpression markedly exacerbated OM-induced calcium deposition in HASMCs, this effect was abrogated by *CREB1* knockdown (Fig. 4D). These observations indicate that CREB1 activation is critical for ADORA2A-mediated osteogenesis and calcification in VSMCs.

### 3.5. ADORA2A promotes VSMC osteogenic differentiation via CREB1-mediated transcriptional enhancement of RUNX2

**Fig. 5.**
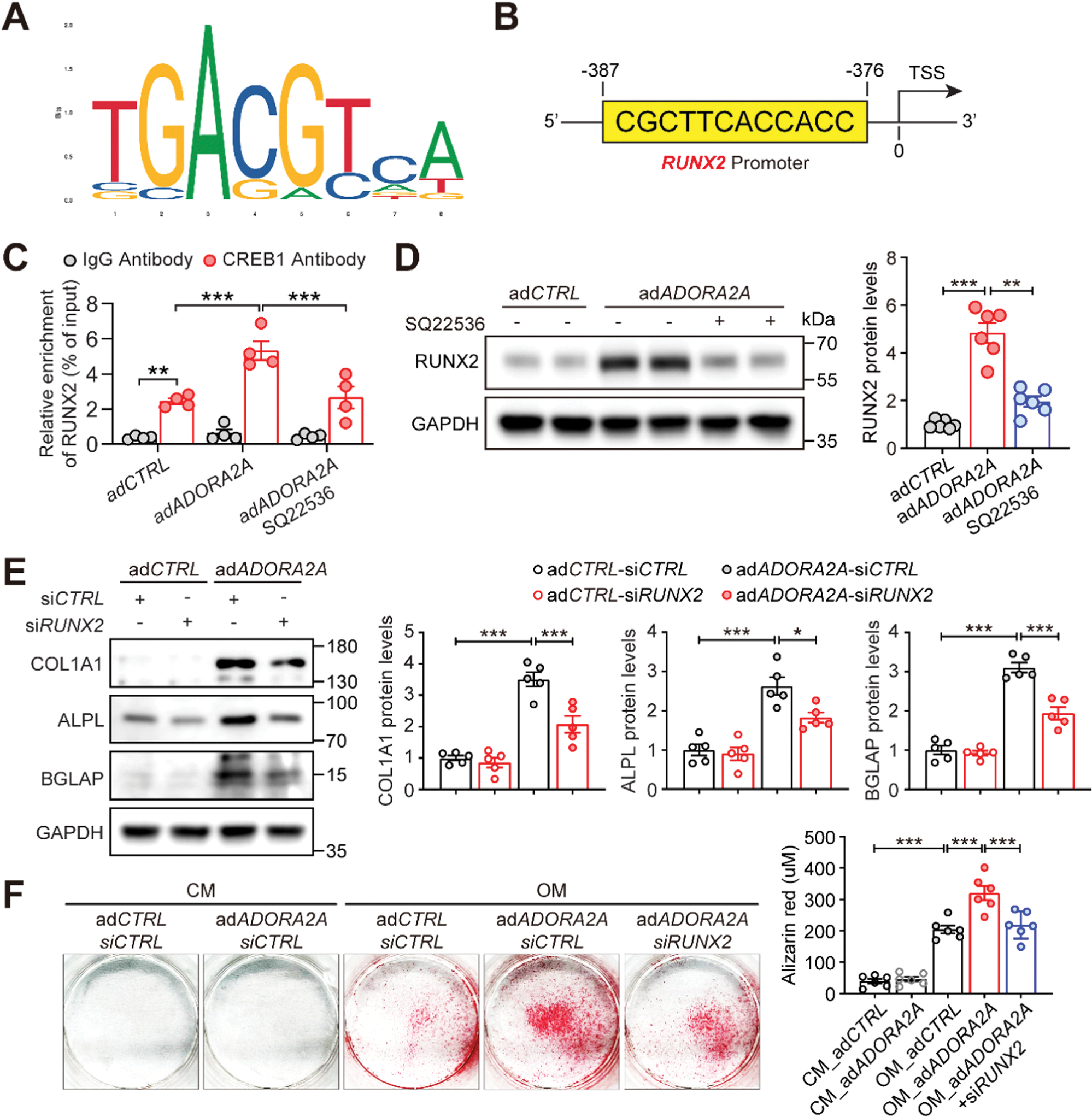
ADORA2A upregulates *RUNX2* transcription via CREB1 to promote VSMC osteogenic differentiation. (A) The JASPAR database predicted the binding domain of CREB1 on the RUNX2 promoter. (B) The location of the binding region of CREB1 on the *RUNX2* promoter was analyzed. (C) ChIP-qPCR analyses of the enrichment of CREB1 on the *RUNX2* promoter in HASMCs treated with vehicle or SQ22536 (10 μM) and infected with control or *ADORA2A* adenovirus for 5 days (n = 4). (D) Western blot analysis and quantification of RUNX2 protein expression in HASMCs treated with vehicle or SQ22536 (10 μM) and infected with control or *ADORA2A* adenovirus for 5 days (n = 6). (E) Western blot analysis and quantification of indicated protein expression in HASMCs transfected with control or *RUNX2* siRNA and infected with control or *ADORA2A* adenovirus for 5 days (n = 5). (F) Representative images and quantification of alizarin red-stained HASMCs transfected with control or *RUNX2* siRNA and infected with control or *ADORA2A* adenovirus, followed by OM treatment for 21 days (n = 6). Data are represented as means ± SEM. Statistical significance was determined by one-way ANOVA with the Bonferroni’s *post hoc* test. *p < 0.05, **p < 0.01, and ***p < 0.001 for indicated comparisons.

To understand how ADORA2A regulates VSMC osteogenic differentiation, we investigated its role in controlling the key transcription factor RUNX2, as demonstrated by the results in Figs. 2 and 3. Bioinformatic analysis revealed a potential binding site of CREB1 on the *RUNX2* promoter (Figs. 5A and 5B). We therefore hypothesized that ADORA2A acts via a CREB1-RUNX2 signaling axis. Our ChIP-qPCR results showed direct binding of CREB1 to the *RUNX2* promoter in VSMCs overexpressing *ADORA2A* (Fig. 5C). This interaction was dependent on the upstream cAMP pathway, as it was significantly weakened by the adenylate cyclase inhibitor SQ22536 (Fig. 5C). In line with these changes in promoter binding, *ADORA2A* overexpression increased RUNX2 at the protein level, and this was abrogated by SQ22536 (Fig. 5D).

To further elucidate the role of RUNX2 in ADORA2A-mediated osteogenesis, we investigated the effects of *RUNX2* knockdown in VSMCs overexpressing *ADORA2A*. *ADORA2A*-overexpressing HASMCs were co-transfected with a small interfering RNA targeting *RUNX2* (si*RUNX2*). Western blot analysis of osteogenic differentiation markers (COL1A1, ALPL, and BGLAP) revealed that *RUNX2* knockdown effectively counteracted the enhanced osteogenic differentiation typically promoted by *ADORA2A* overexpression (Fig. 5E). Furthermore, the impact of *RUNX2* knockdown on mineralization was assessed in HASMCs cultured under OM conditions and concurrently overexpressing *ADORA2A*. Consistent with the findings on osteogenic differentiation, *RUNX2* knockdown resulted in a marked attenuation of the increased calcium deposition that was induced by *ADORA2A* overexpression in this OM-stimulated environment (Fig. 5F). Collectively, these observations suggest that RUNX2 serves as a crucial downstream mediator in the pathway through which ADORA2A promotes osteogenic differentiation and subsequent calcification in VSMCs.

### 3.6. Adora2a deficiency attenuates VC ex vivo and in vivo

**Fig. 6.**
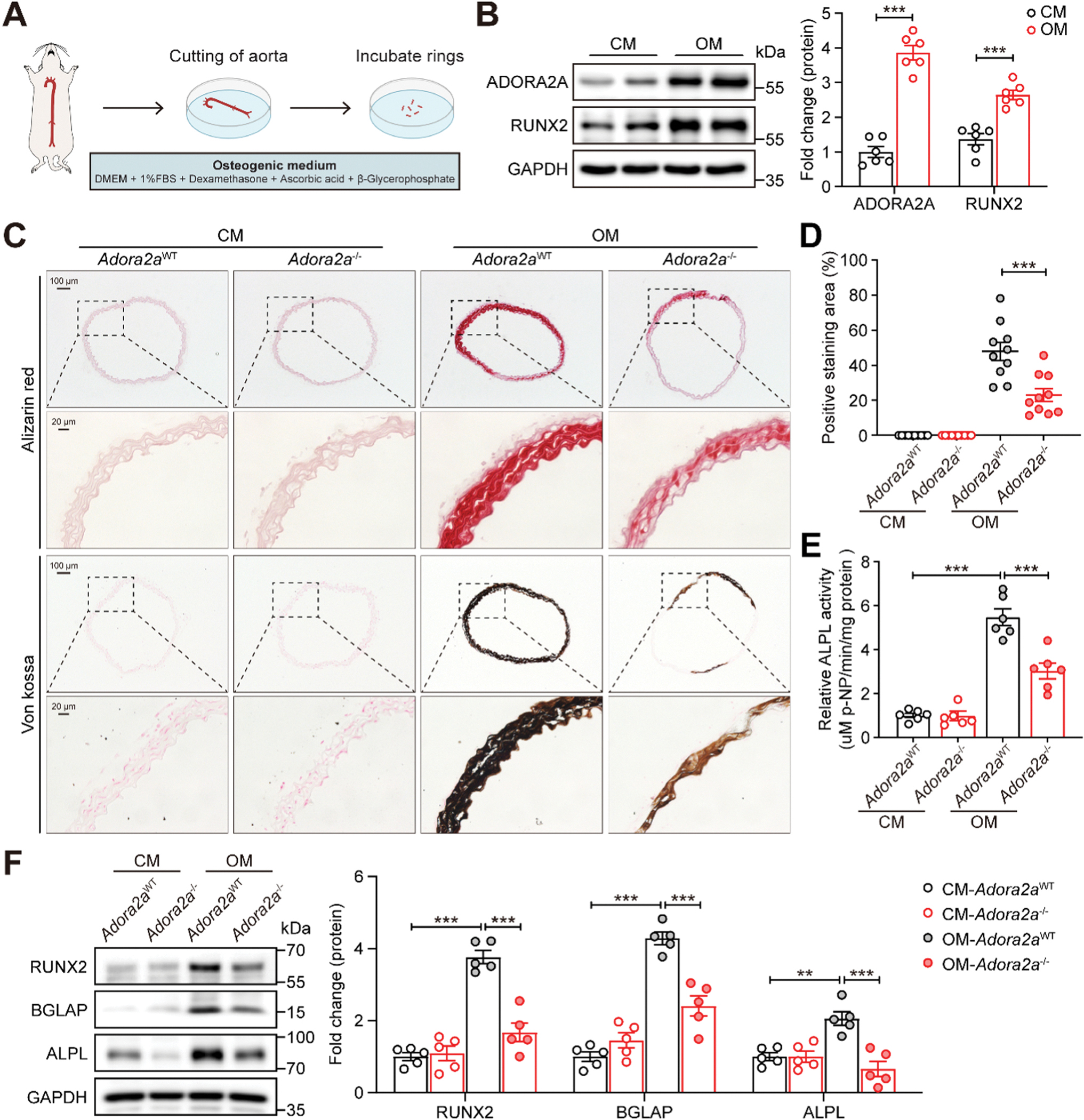
Global *Adora2a* deficiency inhibits *ex vivo* aortic calcification. (A) Schematic of the experimental design for *ex vivo* aortic calcification model. (B) Western blot analysis and quantification of ADORA2A and RUNX2 in aortic rings exposed to OM for 9 days (n = 6). (C) Representative images of alizarin red- and von Kossa-stained aortic sections from aortic rings exposed to OM for 14 days (n = 10). (D) Quantification of calcification in the aortic sections, measured using ImageJ software. The results presented are the percentage of positively stained areas in whole aortic rings (n = 10). (E) Quantification data of ALPL activity in aortic rings exposed to OM for 14 days (n = 6). (F) Western blot analysis and quantification of osteogenic markers in aortic rings exposed to OM for 9 days (n = 5). Data are represented as means ± SEM. Statistical significance was determined by unpaired two-tailed Student’s *t-test* (B and D) and one-way ANOVA with Bonferroni’s *post hoc* test (E and F). **p < 0.01, and ***p < 0.001 for indicated comparisons.

To evaluate ADORA2A as a potential target, we assessed the effect of global *Adora2a* deficiency in VC. Firstly, we harvested descending aortas from *Adora2a*^−/−^ mice and their wild-type *Adora2a*^WT^ littermates to conduct *ex vivo* VC assays by culturing aortic rings in either CM or OM conditions (Fig. 6A). Consistent with observations in human and murine calcified aortic tissues, we found that OM induced ADORA2A expression in the cultured aortas of wild-type mice (Fig. 6B). We further examined calcium deposition in the aorta using alizarin red and von Kossa staining, which revealed that incubation with OM markedly enhanced arterial calcification in aortic rings of *Adora2a*^WT^ mice but not in *Adora2a*^−/−^ mice (Figs. 6C and 6D). ALPL activity was also blunted by *Adora2a* deficiency (Fig. 6E). Meanwhile, the elevated protein levels of osteogenic markers RUNX2, BGLAP, and ALPL were reduced in the aortas of *Adora2a*^−/−^ mice compared with those of *Adora2a*^WT^ mice under osteogenic conditions (Fig. 6F). To determine whether ADORA2A exerts a therapeutic—rather than merely preventive—effect on VC, we isolated descending aortas from C57BL/6 J mice and cultured *ex vivo* under CM or OM conditions for 9 days, followed by treatments with vehicle or KW6002 until day 14. (Supplementary material online, Figure S7A). Alizarin red staining showed the formation of VC on day 9 (Supplementary material online, Figure S7B). Importantly, alizarin red staining on day 14 showed that KW6002 treatment attenuated the progression of *ex vivo* VC compared to vehicle treatment (Supplementary material online, Figure S7C and S7D). This observation supports that pharmacologic ADORA2A blockade slows VC progression and can serve as a therapeutic approach in VC treatment.

**Fig. 7.**
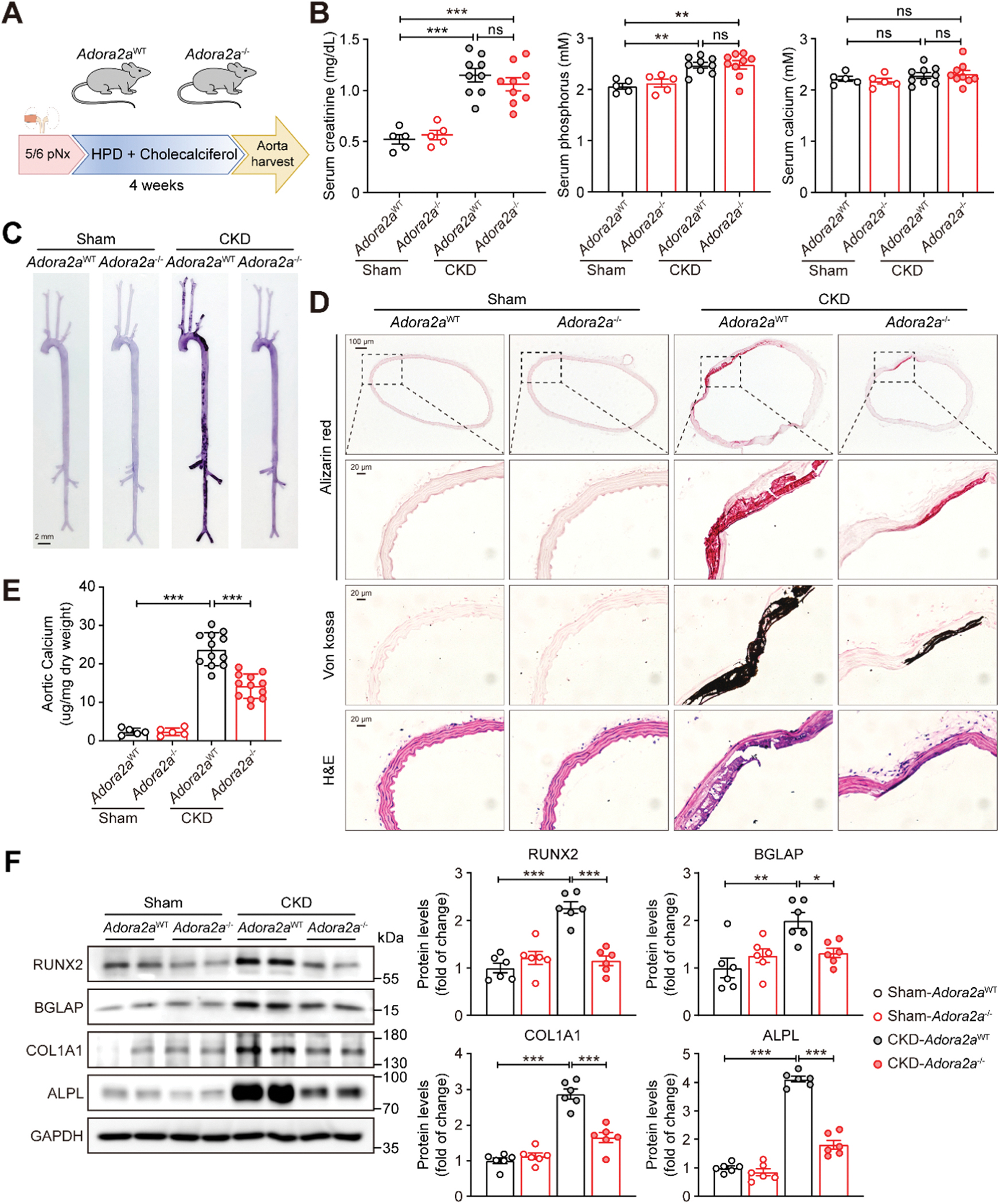
Global *Adora2a* deficiency attenuates VC in CKD mice. (A) Schematic of the experimental design for the CKD-associated VC model in *Adora2a*^WT^ and *Adora2a*^−/−^ mice. (B) Serum concentration of creatinine, phosphorus, and calcium of *Adora2a*^WT^ and *Adora2a*^−/−^ mice (n = 5 for sham group; n = 9 for CKD group). (C) Representative images of alizarin red-stained aortas of *Adora2a*^WT^ and *Adora2a*^−/−^ mice (n = 3). (D) Representative images of alizarin red-, von Kossa-, and HE-stained aortic sections of *Adora2a*^WT^ and *Adora2a*^−/−^ mice (n = 6). (E) Total calcium content in the descending aortas of *Adora2a*^WT^ and *Adora2a*^−/−^ mice. The results shown are normalized by dry weight (n = 5 for the sham group; n = 12 for the CKD group). (F) Western blot analysis and quantification of osteogenic markers in thoracic aortas of *Adora2a*^WT^ and *Adora2a*^−/−^ mice (n = 6). Data are represented as means ± SEM. Statistical significance was determined by one-way ANOVA with the Bonferroni’s *post hoc* test (B and F) and Brown-Forsythe and Welch’s ANOVA test with Dunnett’s T3 multiple comparison test (E). *p < 0.05, **p < 0.01, and ***p < 0.001 for indicated comparisons. “ns” indicates no significant difference.

Moreover, the involvement of ADORA2A in VC was determined *in vivo* by establishing CKD models with *Adora2a*^−/−^ mice and their wild-type littermate controls (Fig. 7A). Serum chemistry analysis revealed that Adora2a deficiency did not alter the elevated levels of serum creatinine and phosphorus in CKD mice, although serum calcium levels remained comparable between the sham and CKD groups (Fig. 7B). We then performed a whole mount of the aorta, staining it with alizarin red, to examine the extent of aortic calcification. As expected, *Adora2a*^−/−^ mice displayed reduced calcification compared to the wild-type controls in the CKD group (Fig. 7C). This result was further confirmed by alizarin red and von Kossa staining of aortic sections and quantification of calcium content in aortas (Figs. 7D and 7E). Histological features of the aortic wall were characterized by the hematoxylin and eosin (HE)-stained aortic sections. The aortas of the sham group exhibited even thickness and normal integrity of elastin fibers throughout the aortic wall. The aortas of the CKD group showed disrupted elastin fiber architecture. In contrast, these abnormal changes in vessel wall structure in CKD mice were mitigated in *Adora2a*^−/−^ mice compared with *Adora2a*^WT^ mice (Fig. 7D). Moreover, the influence of ADORA2A on osteogenic differentiation was assessed using western blot analysis of aortic lysates. In parallel with increased aortic calcification, CKD mice showed enhanced expression of osteogenic markers (RUNX2, BGLAP, COL1A1, and ALPL) in the aortas compared with sham-operated mice; however, this increase was blocked by *Adora2a* deficiency (Fig. 7F).

### 3.7. Pharmacological inhibition of ADORA2A protects against VC in mice

**Fig. 8.**
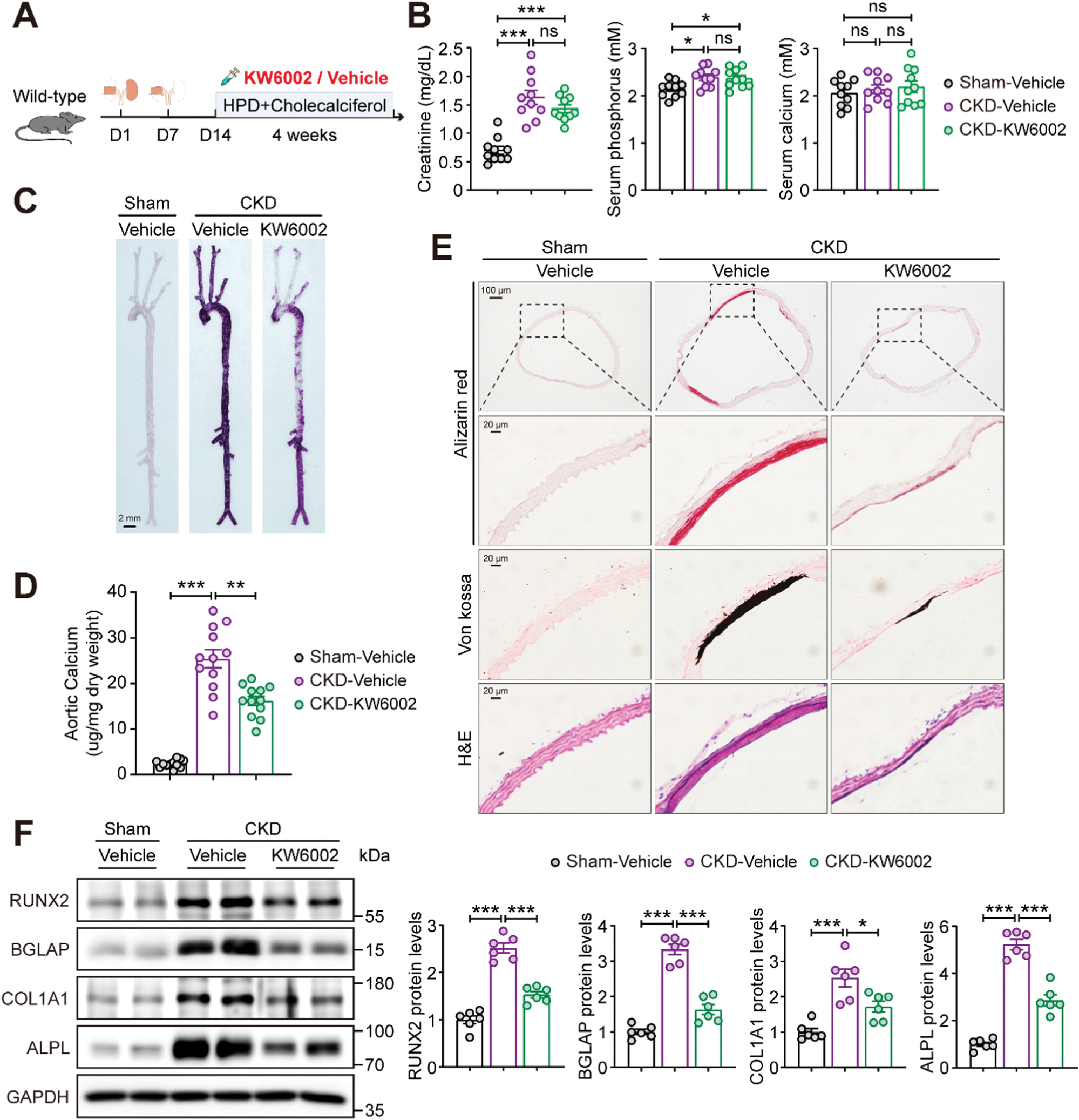
Pharmacological inhibition of ADORA2A with KW6002 protects against VC in CKD mice. (A) Schematic of the experimental design for the CKD-associated VC model in wild-type mice with KW6002 treatment. KW6002 was administered intraperitoneally once daily for 4 weeks, in conjunction with a high-phosphate diet and a cholecalciferol supplement. (B) Serum concentration of creatinine, phosphorus, and calcium of vehicle- and KW6002-treated mice (n = 10). (C) Representative images of alizarin red-stained aortas of vehicle- and KW6002-treated mice (n = 3). (D) Total calcium content in the descending aortas of vehicle- and KW6002-treated mice. Results shown are normalized by dry weight (n = 12). (E) Representative images of alizarin red-, von Kossa- and HE-stained aortic sections of vehicle- and KW6002-treated mice (n = 6). (F) Western blot analysis and quantification of indicated protein expression in thoracic aortas of vehicle- and KW6002-treated mice (n = 6). Data are represented as means ± SEM. Statistical significance was determined by one-way ANOVA with the Bonferroni’s *post hoc* test (B, F) and Brown-Forsythe and Welch’s ANOVA test with Dunnett’s T3 multiple comparison test (D). *p < 0.05, **p < 0.01, and ***p < 0.001 for indicated comparisons. “ns” indicates no significant difference.

In addition to genetic approaches, we also examined whether pharmacological inhibition of ADORA2A reduces VC in CKD mice. KW6002, a selective ADORA2A antagonist, was used for this study. Female DBA/2 mice, a calcification-susceptible mouse strain [29], were randomly assigned to sham, vehicle-treated, and KW6002-treated CKD groups. KW6002 was administered intraperitoneally to mice at a dose of 10 mg/kg daily for four weeks, in conjunction with a high phosphate diet and cholecalciferol supplementation (Fig. 8A). Compared with vehicle treatment, KW6002 administration did not influence the elevated serum creatinine or phosphorus levels in CKD mice; serum calcium levels did not differ among the experimental groups (Fig. 8B). However, KW6002 administration markedly decreased calcification in the whole aortas (Figs. 8C and 8D). Histology of the aortas, as assessed by HE staining, as well as alizarin red and von Kossa staining, also revealed that treatment with KW6002 mitigated the structural disruption of the arterial vessel wall (Fig. 8E) and reduced osteogenic activity compared with vehicle treatment (Fig. 8F). Likewise, in cultured HASMCs, KW6002 treatment resulted in reduced calcium nodule formation and decreased ALPL activity (Supplementary material online, Figure S8A and S8B). KW6002 partially reversed the phenotypic transformation of HASMCs to osteoblast-like cells during the osteogenic differentiation (Supplementary material online, Figure S8C and S8D). These results demonstrate that KW6002 offers an anti-calcification effect in CKD mice by inhibiting osteogenic differentiation.

## 4. Discussion

In this study, we provide compelling evidence that the expression of ADORA2A was markedly elevated in aortic tissues during the progression of calcification in both humans and rodents. Mice with VSMC-specific ablation, global deletion, or pharmacological antagonism of ADORA2A were protected against aortic calcification, as evidenced by a significant reduction of osteogenic differentiation and calcium deposition. Mechanistically, ADORA2A modulated osteogenesis and calcification, at least partially, through the activation of the cAMP-CREB1-RUNX2 axis. Notably, given that tamoxifen induction preceded the occurrence of VC, our inducible *Adora2a* knockout mouse model reflects a preventive (pre-induction) rather than a therapeutic approach. In support of a therapeutic strategy, our *ex vivo* data indicate that pharmacologic ADORA2A blockade slows down the progression of established calcification, further supporting ADORA2A as a druggable target for late intervention. Thus, these findings suggest that ADORA2A is a promising target for VC prevention and, potentially, an avenue for therapeutic intervention. However, a limitation to the generalizability of our study is that it did not consider gender/sex issues.

The role of adenosine signaling in calcification development is complex. The role of adenosine in VC has been extensively studied in patients with arterial calcification due to deficiency of *CD73* (ACDC) [30]. CD73, a membrane protein encoded by the 5′-nucleotidase ecto (*NT5E*) gene, hydrolyzes extracellular adenosine monophosphate (AMP) to produce adenosine under pathological conditions [31]. *NT5E* mutation results in the loss of function of CD73 and decreased extracellular adenosine, leading to increased activity of ALPL [32]. The latter leads to the degradation of pyrophosphate (PPi), a potent calcification inhibitor, into inorganic phosphate (Pi), a stimulator of calcification [30]. In contrast to low adenosine-signaling-caused VC in ACDC patients, adenosine-ADORA2A signaling has been reported to act in diseases with the mechanisms commonly seen in VC development, at least in part. For instance, ADORA2A activation enhances collagen synthesis in fibrotic diseases [33–35]. *Adora2a* deficiency reduces atherosclerotic plaque formation in *Apoe*^−/−^ mice [36] and improves post-stroke outcomes by down-regulating endothelial inflammation [18]. Concordantly, caffeine-driven blockade of the ADORA2A lessened severity in the murine in hypoxia-related vascular damage [37]. In addition, ADORA2A coupled with Gα_s_ promotes adenosine-induced mineralization of valve interstitial cells (VICs) [38]. In calcified aortic tissues of humans and rodents, the upregulated level of *ADORA2A/Adora2a* is the most significant compared to other adenosine receptor subtypes. In line with the pro-mineralizing effect of ADORA2A on VICs, *ADORA2A* knockdown mitigates osteogenic differentiation of VSMCs, thereby reducing subsequent VC. Moreover, ALPL protein levels were also reduced in *ADORA2A*-silenced vascular cells. More studies are needed to explore why *NT5E* mutation-low adenosine induces VC, while *ADORA2A* knockdown reduces VC. The role of adenosine signaling in the development of VC is likely highly associated with adenosine levels, the cellular location of altered adenosine, and the types and levels of adenosine receptors in vascular cells under pathological conditions.

ADORA2A in VSMCs plays a role in the development of VC. Various types of cells have been implicated in the regulation of VC, with VSMCs playing a pivotal role [6]. The phenotypic transition of VSMCs towards an osteoblast-like state represents the principal mechanism contributing to this pathological process. Identifying novel therapeutic targets that can effectively modulate VSMC phenotypic switching remains a critical area of focus in the field of VC. In this study, we investigated the effects of ADORA2A on VC in VSMCs. VSMC-specific *Adora2a* deficiency inhibited osteogenic differentiation and aortic calcification in CKD mice. We further revealed that the protective effect was achieved by reversing the osteogenesis of VSMCs. Thus, ADORA2A in VSMCs serves as an essential regulator in VC. However, the impact of ADORA2A on other types of cells, such as VECs, immune cells, and fibroblast cells, is not excluded in the development of VC. ADORA2A mediates EndMT, inflammasome production in macrophages, and collagen production in fibroblast cells [12,16,35,39]. It has been reported that EndMT, cytokine production, and extracellular matrix remodeling are essential in the development of VC [4,5].

RUNX2 mediates the pro-calcific effects of ADORA2A in VC. As a master regulator of osteogenic differentiation, RUNX2 promotes this process in VSMCs and subsequent matrix mineralization by driving the expression of many osteogenic genes, including *BGLAP*, *COL1A1*, and *ALPL*[40]. Moreover, RUNX2 expression can be further augmented in phenotypically modulated VSMCs, establishing a positive feedback loop that exacerbates the VC process [41,42]. Indeed, VSMC-specific deletion of *Runx2* has been shown to significantly inhibit the development of VC [43,44], underscoring its pivotal role in VSMC osteogenic differentiation and the pathogenesis of VC. In line with these studies, we observed the upregulation of RUNX2 and its downstream targets (BGLAP, COL1A1, and ALPL) in OM-treated human and rodent aortic tissues in our study, which were calcified. Moreover, we showed that ADORA2A inactivation abolished the upregulation of RUNX2 under calcifying conditions both *in vitro* and *in vivo*. Conversely, *ADORA2A* overexpression strongly enhanced the activation of RUNX2 and accelerated osteogenic differentiation and calcification in HASMCs, while *RUNX2* knockdown blocked the pro-calcification effect of *ADORA2A* overexpression.

The cAMP/PKA/CREB1 pathway, a canonical downstream signaling cascade of ADORA2A, plays a pivotal role in the osteogenic differentiation of various cell types [45,46]. CREB1 has been implicated in the pro-calcific effects mediated by low potassium [47] and transforming growth factor-β1 [48]. Furthermore, CREB1 has been shown to activate RUNX2 [49], although the precise underlying mechanisms have remained elusive. Here, we have demonstrated that ADORA2A recruited CREB1 to the *RUNX2* promoter and activated *RUNX2* transcription to trigger VC. This specific mechanistic link between ADORA2A, CREB1, and RUNX2 in VC pathogenesis has not been previously reported. Thus, the current study provides the first evidence that RUNX2 is a crucial mediator of ADORA2A-induced osteogenic differentiation of vascular cells and the consequent VC.

Novel approaches are urgently needed to prevent and treat VC, as the desired therapeutic effects are rarely achieved with current treatments, including dietary control, phosphorus-binding agents, active vitamin D, vitamin K, and calcium mimetics [5,50,51]. VC is a dynamic and regulated process involving gene expression and cellular differentiation [5]. This implies that targeting the essential genes and cells involved in regulating VC might offer a novel strategy to prevent or reverse this condition. Here, our results strongly suggest that ADORA2A holds significant promise as a new target for VC. Over the past few decades, several ADORA2A inhibitors have been developed that exhibit high specificity for ADORA2A over other adenosine receptors [52,53]. KW6002 is the best-studied one and was approved by the U.S. FDA in 2019 to treat Parkinson’s disease (PD) [54]. In clinical application, the use of KW6002 has shown a very consistent safety profile in clinical trials with > 4000 advanced PD patients [55,56]. Notably, potential side effects related to different biological roles of ADORA2A, such as the control of the immune inflammatory system [11], sleep [57], and vascular tone [58], are not evident in clinical trials, which noted the absence of insomnia, hypertension, or increased infection rates [55,56, 59]. The general lack of side effects of KW6002 and its efficacy in treating VC in rodent models underscores the importance of further investigating the impact of ADORA2A inhibitors in patients with VC.

In summary, our study, for the first time, demonstrates that the inactivation of ADORA2A inhibits osteogenic differentiation of VSMCs and protects against VC through the downregulation of RUNX2 transcriptional activity via the cAMP/PKA/CREB1 signaling pathway. These findings highlight the potential of ADORA2A as a new target for the prevention and therapy of VC, providing a strong foundation for innovative avenues in the prevention and management of CVD in patients with CKD.

## Supplementary Material

Supplementary Material

Figures S1 to S8 for multiple supplementary figures.

Tables S1 to S4 for multiple supplementary tables.

Supplementary figures of uncropped scans for WB.

## Data Availability

Data will be made available on request.

## Appendix A. Supporting information

Supplementary data associated with this article can be found in the online version at doi:10.1016/j.phrs.2025.108012.

## Abbreviations:

CVD: cardiovascular disease
CKD: chronic kidney disease
CLI: critical limb ischemia
ADORA2A: Adenosine 2 A receptor
VC: vascular calcification
VSMCs: vascular smooth muscle cells
HASMCs: human aortic smooth muscle cells
ARs: adenosine receptors
GPCRs: G protein-coupled receptors
BMPs: bone morphogenetic proteins
RUNX2: runt-related transcription factor 2
BGLAP: osteocalcin
COL1A1: collagen type I alpha 1
ALPL: alkaline phosphatase
TAGLN: smooth muscle 22-alpha
ACTA2: alpha-smooth muscle actin
CREB1: cAMP-responsive element-binding protein 1
