# Supplementary material for: Targeting adenosine 2A receptor signaling suppresses vascular calcification by restraining smooth muscle osteogenic differentiation
Source: Pharmacol Res. Author manuscript; Available in PMC 2026 Jul 3. (PMC13331241; doi:10.1016/j.phrs.2025.108012)
Supplement: Supplementary Material [file NIHMS2137935-supplement-Supplementary_Material.docx]

**SUPPLEMENTAL MATERIALS**

**Targeting adenosine 2A receptor signaling suppresses vascular calcification by restraining smooth muscle osteogenic differentiation**

Yaqi Zhou^a, b^, Dingwei Zhao^b, c^, Qian Ma^c^, Sujin Lee^d^, Kangsan Roh^d^, Yongfeng Cai^c^, Jiean Xu^a,e^, Qiuhua Yang^f^, Qingen Da^b^, Zhiping Liu^e^, Kunfu Ouyang^b^, Eric J Belin de Chantemele^e^, Mei Hong^b^, Clint L. Miller^g, h^, Rajeev Malhotra^d^, Chunxiang Zhang^i,*^, Suowen Xu ^j, k, l*^, and Yuqing Huo^c,*^

^a^Department of Physiology, Research Center of Basic Integrative Medicine, School of Basic Medical Sciences, Guangzhou University of Chinese Medicine, Guangzhou 510006, China

^b^State Key Laboratory of Chemical Oncogenomics, Key Laboratory of Chemical Genomics, School of Chemical Biology and Biotechnology, Peking University Shenzhen Graduate School, Shenzhen 518055, China

^c^Departments of Ophthalmology, Medicine and Molecular and Cellular Biology, Baylor College of Medicine, Houston, TX 77030, USA

^d^Cardiovascular Research Center, Division of Cardiology, Department of Medicine, Massachusetts General Hospital, Harvard Medical School, Boston, MA 02114, USA

^e^Vascular Biology Center, Department of Cellular Biology and Anatomy, Medical College of Georgia, Augusta University, Augusta, GA 30912, USA

^f^Department of Pharmacological Sciences, Stony Brook University, Stony Brook, NY 11794, USA

^g^Department of Biochemistry and Molecular Genetics, University of Virginia, Charlottesville, VA 22908, USA

^h^Center for Public Health Genomics, University of Virginia, Charlottesville, VA 22908, USA

^i^Department of Cardiology, Key Laboratory of Medical Electrophysiology, Ministry of Education, Institute of Cardiovascular Research, The Affiliated Hospital of Southwest Medical University, Southwest Medical University, Luzhou 646000, China

^j^Department of Endocrinology, Centre for Leading Medicine and Advanced Technologies of IHM, The First Affiliated Hospital of USTC, Division of Life Sciences and Medicine, University of Science and Technology of China, Hefei, 230001, China

^k^Anhui Provincial Key Laboratory of Metabolic Health and Panvascular Diseases, Hefei, 230001, China

^l^Institute of Endocrine and Metabolic Diseases, University of Science and Technology of China, Hefei, 230001, China

^*^**Correspondence:**

Yuqing Huo, MD, PhD

Departments of Ophthalmology, Medicine, and Molecular and Cellular Biology, Baylor College of Medicine, Houston, TX 77030, USA

Phone: +1 713-798-1912 (Office)

Email: [yuqing.huo@bcm.edu](mailto:yuqing.huo@bcm.edu)

Suowen Xu, PhD

Department of Endocrinology, Centre for Leading Medicine and Advanced Technologies of IHM, The First Affiliated Hospital of USTC, Division of Life Sciences and Medicine, University of Science and Technology of China, Hefei, 230001, China

Phone: +86 0551-63602683 (Office)

Email: [sxu1984@ustc.edu.cn](mailto:sxu1984@ustc.edu.cn)

Chunxiang Zhang, MD, PhD

Department of Cardiology, Key Laboratory of Medical Electrophysiology, Ministry of Education, Institute of Cardiovascular Research, The Affiliated Hospital of Southwest Medical University, Southwest Medical University, Luzhou 646000, China

Phone: +86 0830-3161658 (Office)

Email: [zhangchx999@163.com](mailto:zhangchx999@163.com)

**
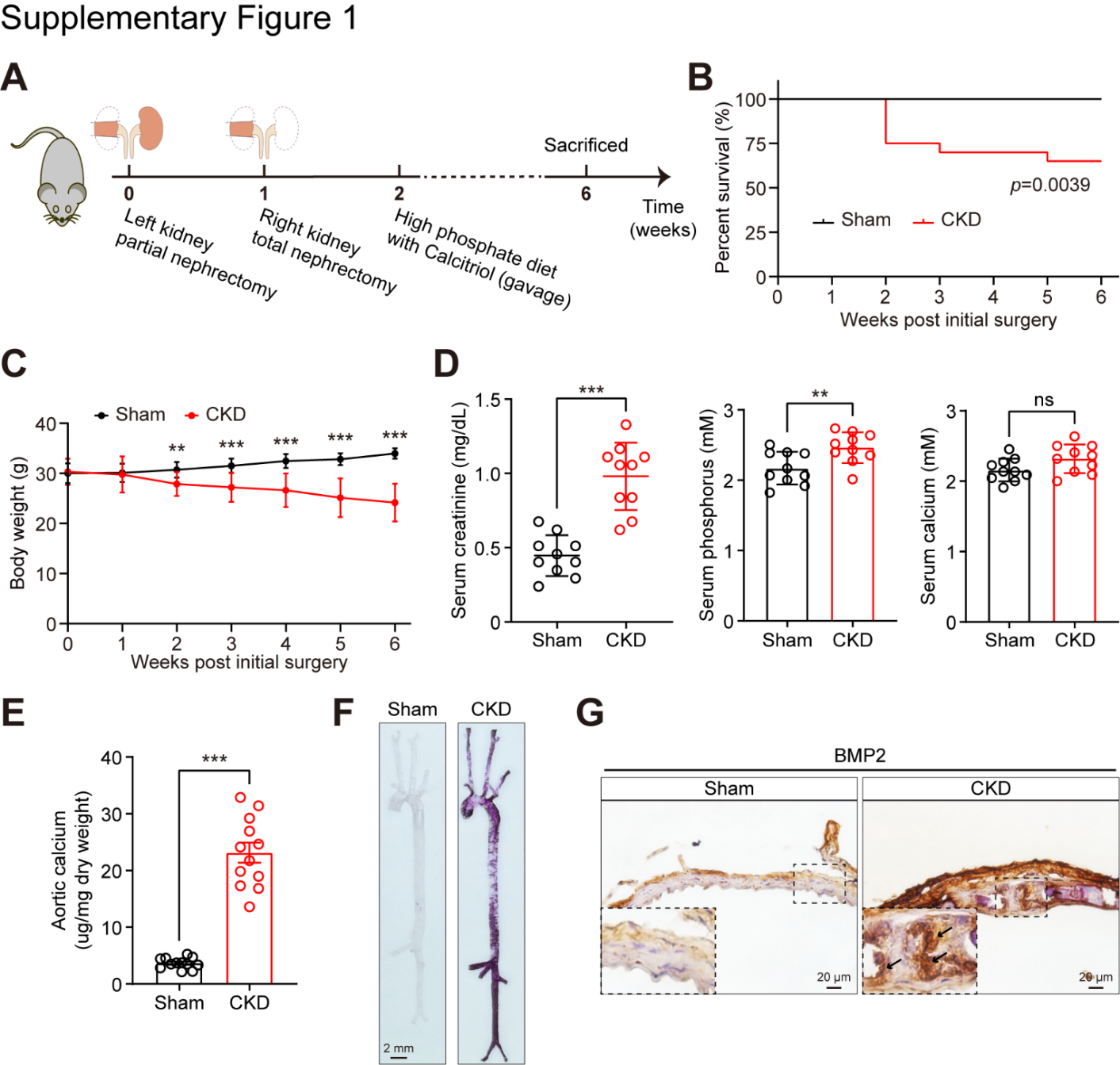
Supplementary Figures and Figure Legends**

**Figure S1. Vascular calcification in sham and CKD mice.** (A) Schematic of the experimental design for the CKD-associated VC model in mice. (B) Survival curve for mice following a 5/6^th^ nephrectomy (n = 20). (C) Body weights in sham and CKD mice (n = 12). (D) Serum concentration of creatinine, phosphorus, and calcium of sham and CKD mice (n = 10). (E) Total calcium content in the descending aortas of sham and CKD mice. Results shown are normalized by dry weight (n = 12). (F) Representative images of alizarin red-stained aortic arteries of sham and CKD mice (n = 4). (G) Representative immunohistochemical staining of BMP2 on the aortic sections of sham and CKD mice (n = 8). Data are represented as mean ± SEM. Statistical significance was determined by the Mantel-Cox test for (B) and unpaired two-tailed Student’s *t*-test for (C-E). ***p* < 0.01, and ****p* < 0.001 for indicated comparisons. **
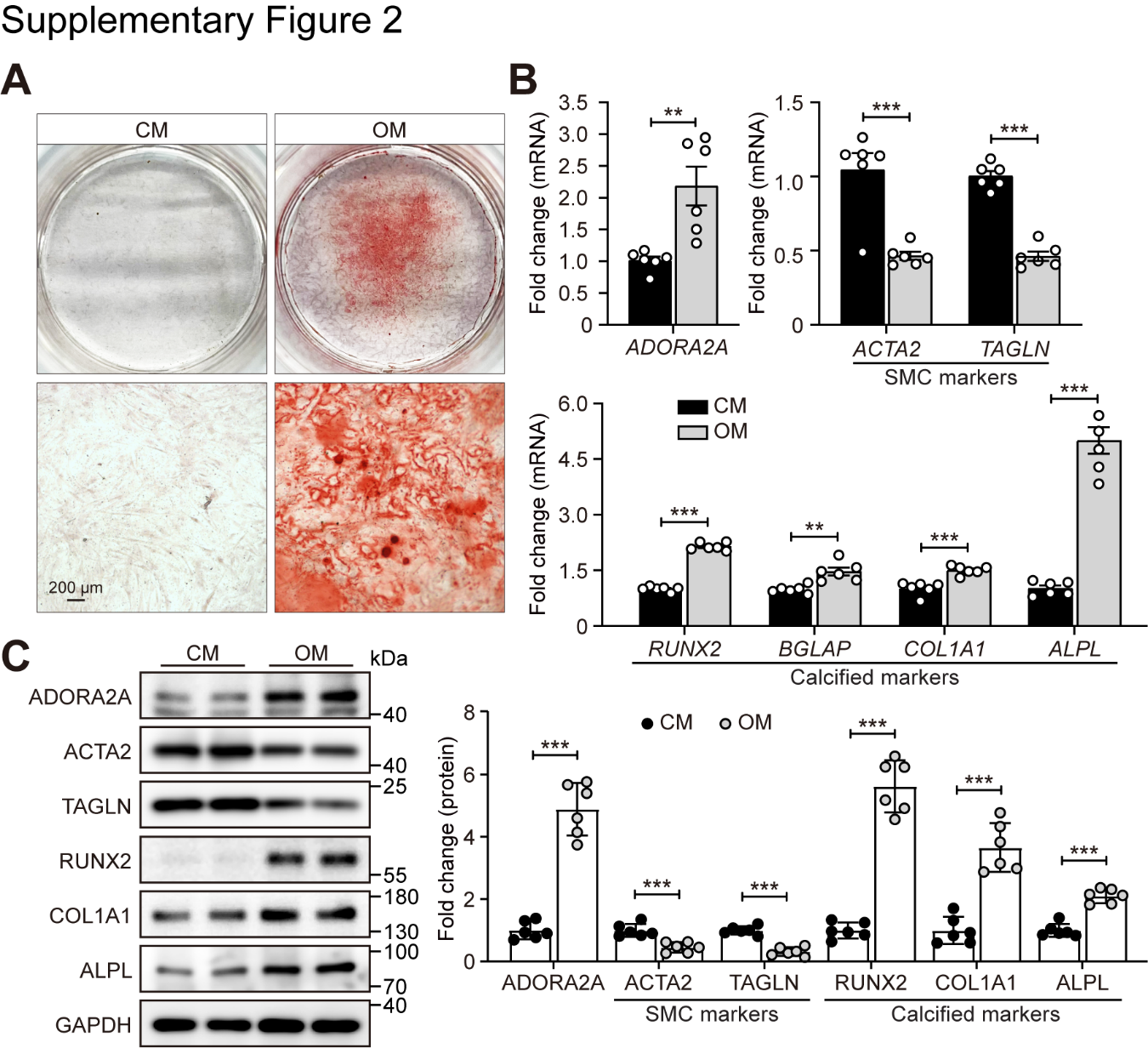
**

**Figure S2. ADORA2A expression in HASMCs under osteogenic conditions.** (A) Representative images of alizarin red staining of HASMCs exposed to OM for 21 days (n = 6). (B) qPCR analysis of mRNA levels of indicated genes in HASMCs exposed to OM for 7 days (n = 6). (C) Western blot analysis and quantification data of indicated protein expression in HASMCs exposed to OM for 7 days (n = 6). Data are represented as mean ± SEM. Statistical significance was determined by unpaired two-tailed Student’s *t*-test. ***p* < 0.01 and ****p* < 0.001 for indicated comparisons.

**
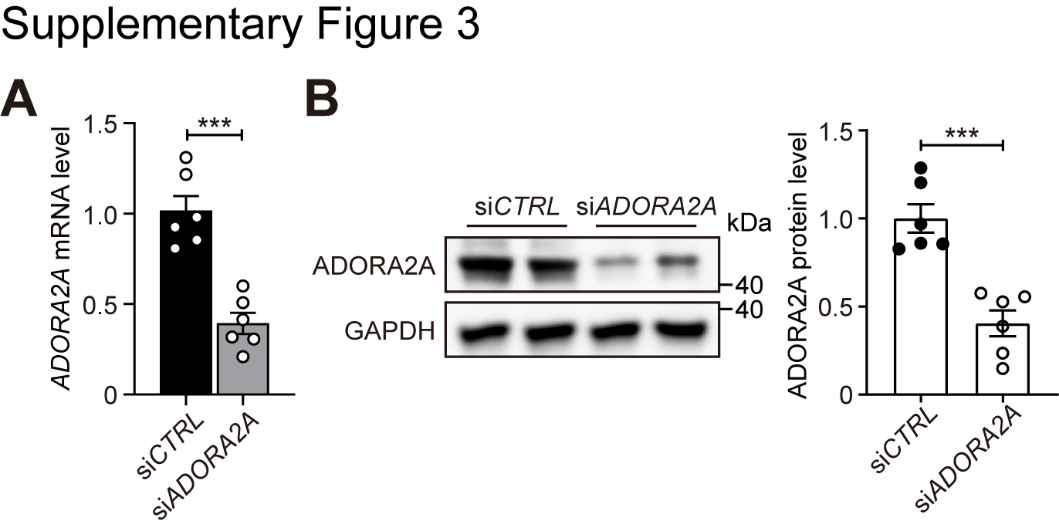
**

**Figure S3. ADORA2A expression in HASMCs with** ***ADORA2A* siRNA transfection.** qPCR analysis (A) and western blot analysis and quantification data (B) of ADORA2A expression in HASMCs transfected with control or *ADORA2A* siRNA for 48 hours (n = 6). Data are represented as mean ± SEM. Statistical significance was determined by unpaired two-tailed Student’s *t*-test. ****p* < 0.001 for indicated comparisons.

**
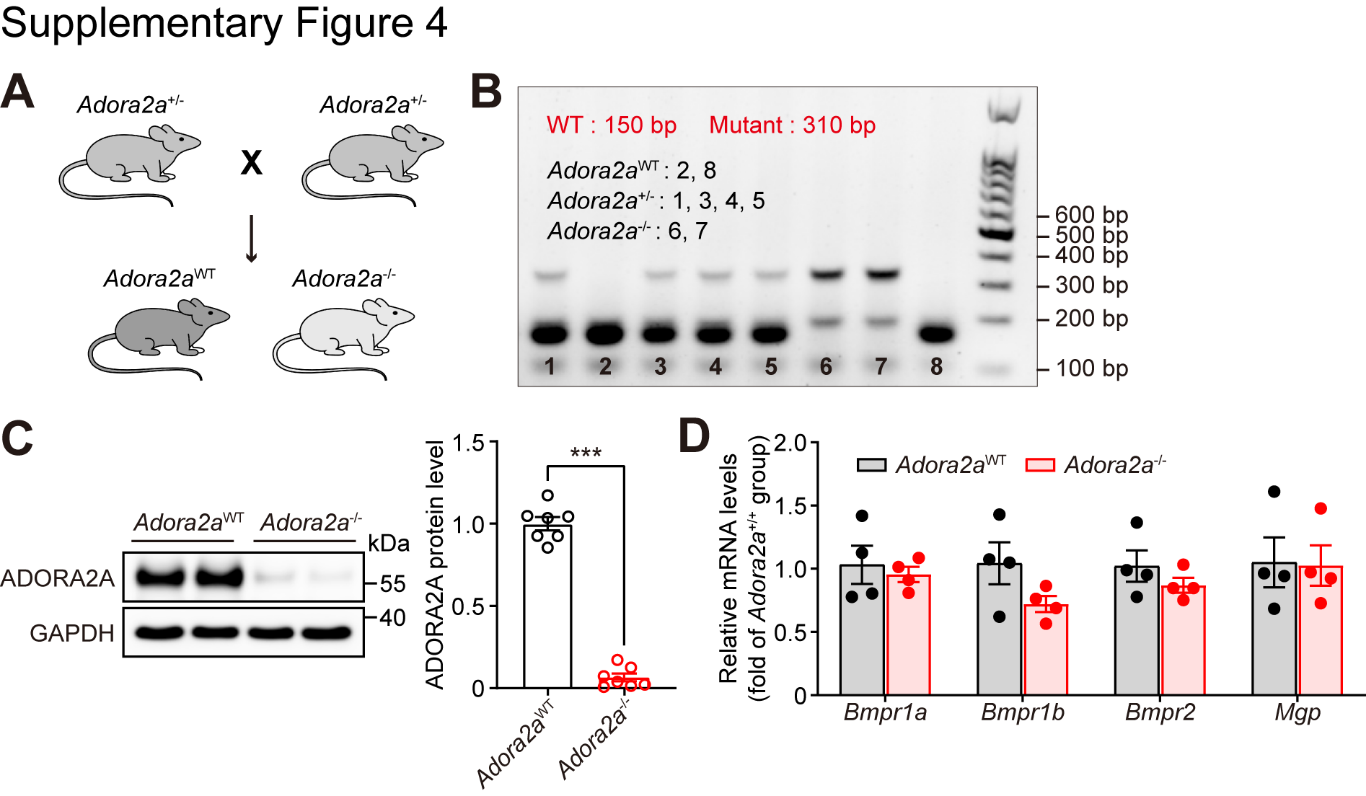
**

**Figure S4. Generation of *Adora2a* global-deficient mice.** (A) Strategy for generating *Adora2a*^-/-^ mice by heterozygous inter-breeding (*Adora2a*^+/-^). (B) Representative genotyping PCR results of *Adora2a*^-/-^, *Adora2a*^+/-^ and *Adora2a*^WT^ mice. (C) Western blot analysis and quantification data of ADORA2A expression in MASMCs from *Adora2a*^-/-^ and *Adora2a*^WT^ mice (n = 4). (D) qPCR analysis of mRNA levels of indicated genes in MASMCs of *Adora2a*^-/-^ and *Adora2a*^WT^ mice (n = 4). Data are represented as mean ± SEM. Unpaired two-tailed Student’s *t*-test determined statistical significance. ****p* < 0.001 for indicated comparisons.
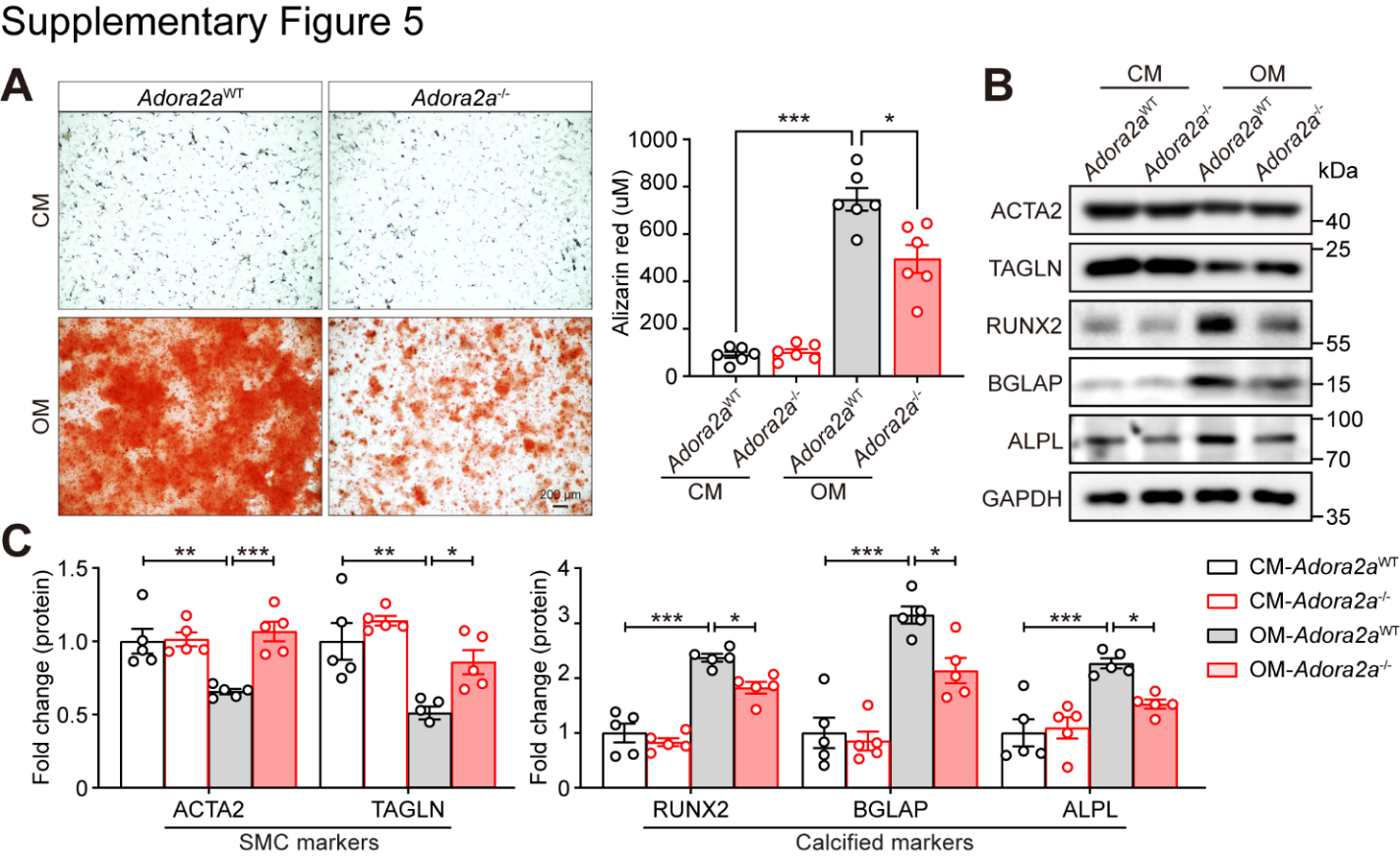


**Figure S5. ADORA2A regulates osteogenic differentiation and calcification of MASMCs.** (A) Representative images and quantification of alizarin red staining on MASMCs isolated from *Adora2a*^-/-^ and *Adora2a*^WT^ mice and exposed to OM for 21 days (n = 6). (B-C) Western blot analysis and quantification data of the indicated protein expression in MASMCs isolated from *Adora2a*^-/-^ and *Adora2a*^WT^ mice, which were exposed to OM for 7 days (n = 5), are presented. Data are represented as mean ± SEM. Statistical significance was determined by Brown-Forsythe and Welch’s ANOVA test with Dunnett’s T3 multiple comparison test (A) and one-way ANOVA with the Bonferroni’s *post hoc* test (C). **p* < 0.05, ***p* < 0.01, and ****p* < 0.001 for indicated comparisons.
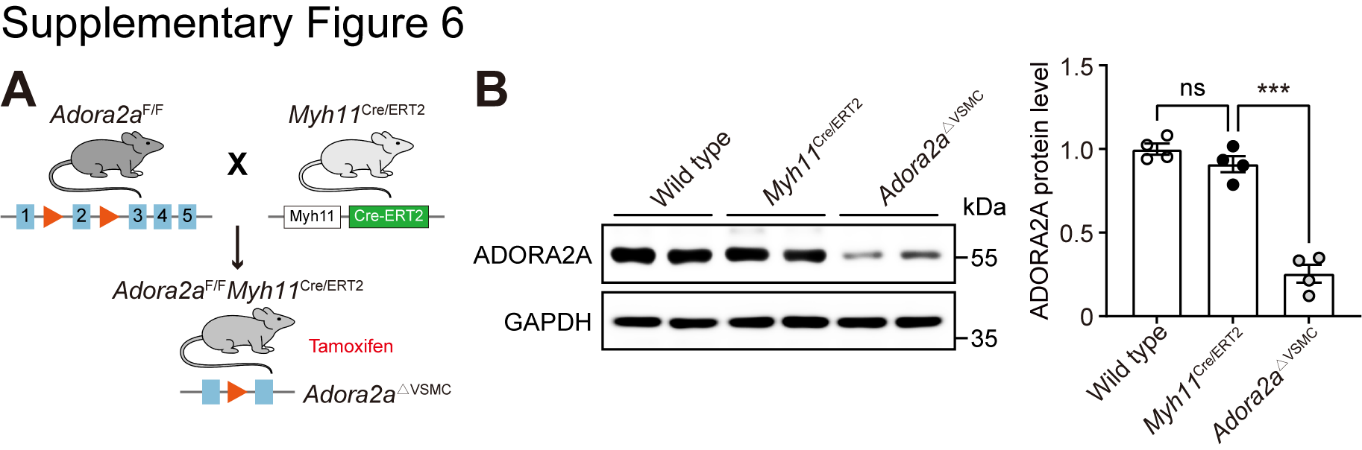


**Figure S6. Generation of VSMC-specific *Adora2a-deficient* mice.** (A) Strategy for generating *Adora2a*^∆VSMC^ mice by crossing *Adora2a*^F/F^ mice with *Myh11*^Cre/ERT2^ mice and tamoxifen treatment of the consequent mice. (B) Western blot analysis and quantification data of ADORA2A expression in media of aortas from *Myh11*^Cre/ERT2^ and *Adora2a*^∆VSMC^ mice (n = 4). Data are represented as mean ± SEM. Statistical significance was determined by one-way ANOVA with Bonferroni’s *post hoc* test (B). ****p* < 0.001 for indicated comparisons. “ns” indicates no significant difference.


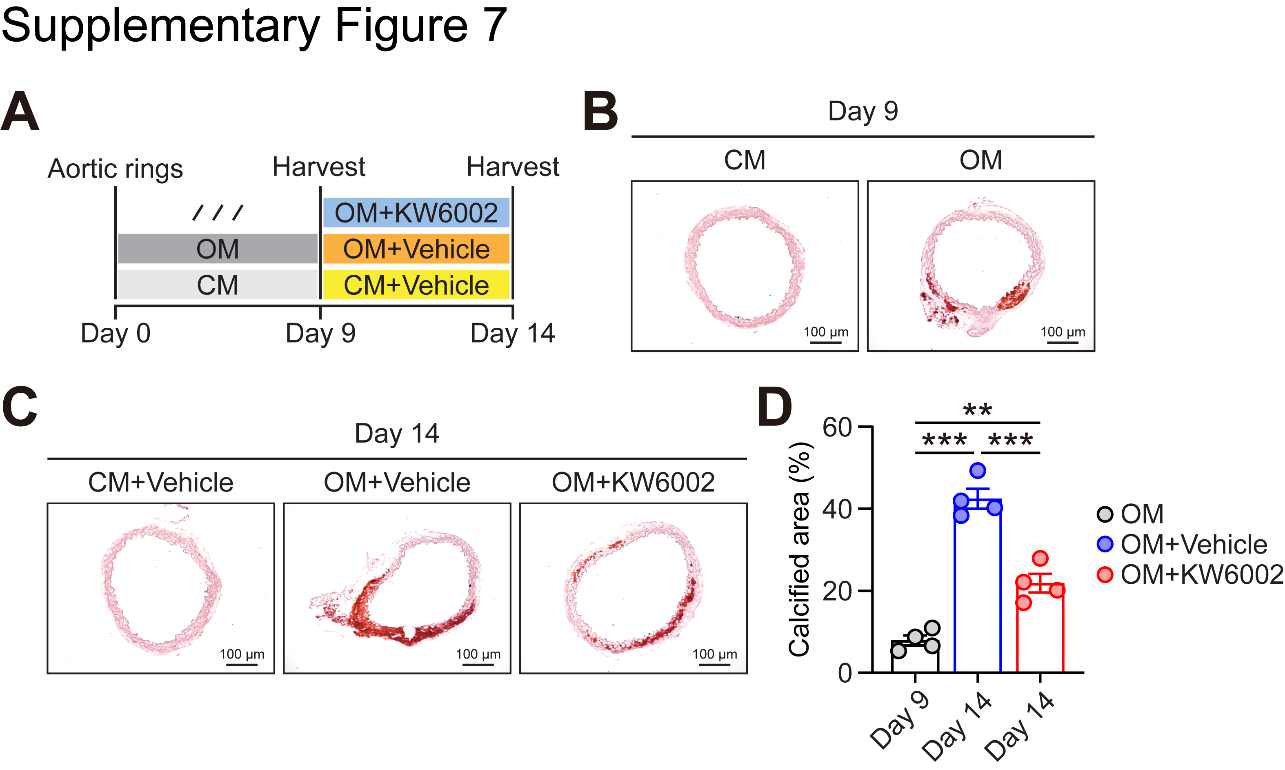


**Figure S7. KW6002 slows down the progression of *ex vivo* aortic calcification.** (A) Schematic of the therapeutic KW6002 effect on the *ex vivo* VC model. (B) Representative images of alizarin red-stained aortic sections from aortic rings exposed to OM for 9 days (CM, n = 2; OM, n = 4). (C) Representative images of alizarin red-stained aortic sections from aortic rings exposed to OM+vehicle or OM+KW6002 for five more days after initial 9-day OM treatment (CM+Vehicle, n = 2; OM+Vehicle, n = 4; OM+KW6002, n = 4). (D) Quantification of calcification in the aortic sections, measured using ImageJ software. The results presented are the percentage of positively stained areas in whole aortic rings (n = 4). Data are represented as mean ± SEM. Statistical significance was determined by ordinary one-way ANOVA with Bonferroni’s *post hoc* test (D). ***p* < 0.01, and ****p* < 0.001 for indicated comparisons.


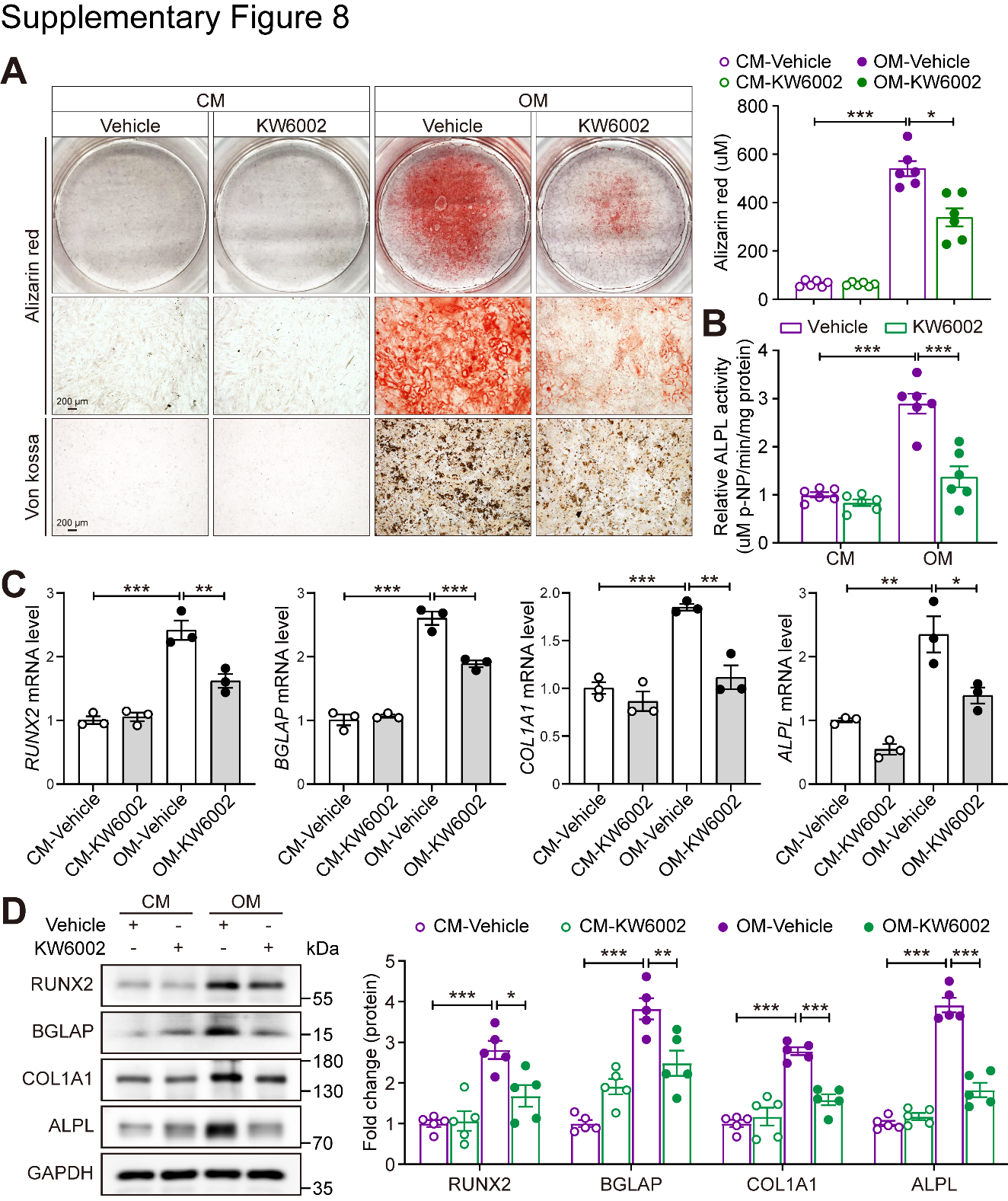


**Figure S8. KW6002 prevents osteogenic differentiation and calcification of HASMCs.** (A) Representative images and quantification of alizarin red and von Kossa staining on HASMCs treated with vehicle or KW6002 and exposed to OM for 21 days (n = 6). (B) Quantification data of ALPL activity of HASMCs treated with vehicle or KW6002 and exposed to OM for 14 days (n = 6). (C) qPCR analysis of mRNA levels of indicated genes in HASMCs treated with vehicle or KW6002 and exposed to OM for 7 days (n = 3). (D) Western blot analysis and quantification data of indicated protein expression in HASMCs treated with vehicle or KW6002 and exposed to OM for 7 days (n = 5). Data are represented as means ± SEM. Statistical significance was determined by Brown-Forsythe and Welch's ANOVA test with Dunnett's T3 multiple comparison test (A) and one-way ANOVA with the Bonferroni’s *post hoc* test (B-D). **p* < 0.05, ***p* < 0.01, and ****p* < 0.001 for indicated comparisons.**Supplementary Tables**

**Supplementary Table 1. Primers used for genotyping of murine strains.**

| **Primers** | **Sequence (5’-3’)** | **Product size** |
| --- | --- | --- |
| *Adora2a flox* | GGGCAAGATGGGAGTCATT | WT: 180bp  Mutant: 220bp |
|  | ATTCTGCATCTCCCGAAACC |  |
| *Myh11*^Cre/ERT2^ | TGACCCCATCTCTTCACTCC | WT: 180bp  Mutant: 287bp |
|  | AGTCCCTCACATCCTCAGGTT |  |
| *Myh11*^WT^ | CAGCCAACTTTACGCCTAGC |  |
|  | TCTCAAGATGGACCTAATACGG |  |
| *Adora2a^-/-^* | AGCCAGgggttacatctgtg | WT: 150bp  Mutant: 310bp |
|  | tacagacagcctcgacatgtg |  |
|  | tcggccattgaacaagatgg |  |
|  | gagcaaggtgagatgagagg |  |

**Supplementary Table 2. Antibodies used for western blot.**

| **Antibodies** | **Working concentration** | **Catalog No.** | **Manufacturers** |
| --- | --- | --- | --- |
| Anti-ADORA2A | 1 μg/mL | 05-717 | Sigma-Aldrich |
| Anti-RUNX2 | 1:1000 | 12556 | Cell Signaling Technology |
| Anti-BGLAP | 1 μg/mL | ab93876 | Abcam |
| Anti-COL1A1 | 1 μg/mL | NB600408 | Novus Biologicals |
| Anti-ALPL | 0.55 μg/mL | 11187-1-AP | ProteinTech |
| Anti-ACTA2 | 0.1 μg/mL | sc-56499 | Santa Cruz Biotechnology |
| Anti-TAGLN | 0.5 μg/mL | ab14106 | Abcam |
| Anti-CREB1 | 1:1000 | 9197S | Cell Signaling Technology |
| Anti-p-CREB1 (Ser133) | 1:1000 | 9198S | Cell Signaling Technology |
| Anti-GAPDH | 1:3000 | 5174S | Cell Signaling Technology |
| Anti-rabbit IgG, HRP-linked antibody | 1:5000 | 7074 | Cell Signaling Technology |
| Anti-mouse IgG, HRP-linked antibody | 1:5000 | 7076 | Cell Signaling Technology |

**Supplementary Table 3. Primers used for Quantitative RT-PCR.**

| **Gene** | **Forward (5’-3’)** | **Reverse (5’-3’)** |
| --- | --- | --- |
| Murine *Adora2a* | TCCACTCCGGTACAATGGCTT | TGACTGCAGTTGTTCCAGCCC |
| Murine *Bmpr1a* | AACAGCGATGAATGTCTTCGAG | GTCTGGAGGCTGGATTATGGG |
| Murine *Bmpr1b* | CCTCGGCCCAAGATCCTAC | CCTAGACATCCAGAGGTGACA |
| Murine *Bmpr2* | TTGGGATAGGTGAGAGTCGAAT | TGTTTCACAAGATTGATGTCCCC |
| Murine *Acta* | TGTCCACCTTCCAGCAGATGT | AGCTCAGTAACAGTCCGCCTAG |
| Human *ADORA2A* | CGAGGGCTAAGGGCATCATTG | CTCCTTTGGCTGACCGCAGTT |
| Human *ACTA2* | TCCGCTTCAATTCCTGTCCG | CTTGATGCGAAGTGCTGACC |
| Human *TAGLN* | AGTGCAGTCCAAAATCGAGAAG | CTTGCTCAGAATCACGCCAT |
| Human *RUNX2* | TAGGCGCATTTCAGGTGCTT | GACATGCCTGAGGTGACTGG |
| Human *BGLAP* | GGCGCTACCTGTATCAATGG | GTGGTCAGCCAACTCGTCA |
| Human *COL1A1* | GTGCGATGACGTGATCTGTGA | CGGTGGTTTCTTGGTCGGT |
| Human *ALPL* | ACTGGTACTCAGACAACGAGAT | ACGTCAATGTCCCTGATGTTATG |
| Human *ACTA* | CATGTACGTTGCTATCCAGGC | CTCCTTAATGTCACGCACGAT |
| 18S rRNA | CTTAGAGGGACAAGTGGCG | ACGCTGAGCCAGTCAGTGTA |

**Supplementary Table 4. Raw FPKM values for adenosine receptors in individual samples from Control and CKD Grou****ps (****GSE159833)**

| **Gene Name** | **PFKM** | | | | **Mean Fold Change** | **Log_2_ (Fold Change)** |
| --- | --- | --- | --- | --- | --- | --- |
|  | **Ctrl-1** | **Ctrl-2** | **CKD-1** | **CKD-2** |  |  |
| *Adora1* | 0.603 | 1.587 | 2.549 | 1.520 | 1.858 | 0.894 |
| *Adora2a* | 0.981 | 0.906 | 2.430 | 1.764 | 2.222 | 1.152 |
| *Adora2b* | 0.261 | 0.358 | 0.293 | 0.600 | 1.445 | 0.531 |
| *Adora3* | 0.048 | 0.035 | 0 | 0.056 | 0.665 | -0.589 |
